# N6-Methyladenosine-Related LncRNAs Are Potential Remodeling Indicators in the Tumor Microenvironment and Prognostic Markers in Osteosarcoma

**DOI:** 10.3389/fimmu.2021.806189

**Published:** 2022-01-12

**Authors:** Zhongguang Wu, Xiaobo Zhang, Dongjie Chen, Zian Li, Xin Wu, Jianlong Wang, Youwen Deng

**Affiliations:** ^1^ Department of Laboratory Medicine, The Third Xiangya Hospital, Central South University, Changsha, China; ^2^ Department of Spine Surgery, Third Xiangya Hospital, Central South University, Changsha, China; ^3^ Department of Hepatopancreatobiliary Surgery, The Third Xiangya Hospital, Central South University, Changsha, China; ^4^ Department of Clinical Laboratory, Qinghai Provincial People’s Hospital, Xining, China

**Keywords:** epigenetic, N6-methyladenosine (m6A), osteosarcoma, plasma cell, long non-coding RNAs (lncRNAs), tumor microenvironment

## Abstract

N6-Adenosine methylation, yielding N6-methyladenosine (m^6^A), is a reversible epigenetic modification found in messenger RNAs and long non-coding RNAs (lncRNAs), which affects the fate of modified RNA molecules and is essential for the development and differentiation of immune cells in the tumor microenvironment (TME). Osteosarcoma (OS) is the most common primary bone tumor in children and adolescents, and is characterized by high mortality. Currently, the possible role of m^6^A modifications in the prognosis of OS is unclear. In the present study, we investigated the correlation between m^6^A-related lncRNA expression and the clinical outcomes of OS patients *via* a comprehensive analysis. Clinical and workflow-type data were obtained from the Genotype-Tissue Expression Program and The Cancer Genome Atlas. We examined the relationship between m^6^A modifications and lncRNA expression, conducted Kyoto Encyclopedia of Genes analysis and also gene set enrichment analysis (GSEA), implemented survival analysis to investigate the association of clinical survival data with the expression of m^6^A-related lncRNAs, and utilized Lasso regression to model the prognosis of OS. Furthermore, we performed immune correlation analysis and TME differential analysis to investigate the infiltration levels of immune cells and their relationship with clinical prognosis. LncRNA expression and m^6^A levels were closely associated in co-expression analysis. The expression of m^6^A-related lncRNAs was quite low in tumor tissues; this appeared to be a predicting factor of OS in a prognostic model, independent of other clinical features. The NOD-like receptor signaling pathway was the most significantly enriched pathway in GSEA. In tumor tissues, *SPAG4* was overexpressed while *ZBTB32* and *DEPTOR* were downregulated. Tissues in cluster 2 were highly infiltrated by plasma cells. Cluster 2 presented higher ESTIMATE scores and stromal scores, showing a lower tumor cell purity in the TME. In conclusion, m^6^A-related lncRNA expression is strongly associated with the occurrence and development of OS, and can be used to as a prognostic factor of OS. Moreover, m^6^A-related lncRNAs and infiltrating immune cells in the TME could serve as new therapeutic targets and prognostic biomarkers for OS.

## Introduction

Epigenetic silencing, for instance *via* histone modifications or RNA methylation, is a crucial tumorigenic mechanism. In fact, epigenomic alterations affect tumor immunogenicity, and immune cells are involved in antitumor responses (1). Furthermore, epigenetic reprogramming *via* inhibition of methylation enhances plasma cell polarization and decreases the response efficiency of antitumorigenic T cells (2). Cell differentiation, immunity, inflammation, and carcinogenesis are all regulated by non-coding RNAs (ncRNAs) such as microRNAs (miRNAs), long non-coding RNAs (lncRNAs), and circular RNAs (circRNAs) (3). Recent epigenetic studies have focused on N6-methyladenosine (m^6^A). To develop effective antitumor treatments and predict individual survival and recurrence risks, it is essential to unravel the role of m^6^A-related lncRNAs in the development and differentiation of immune cells.

Among the most common invasive bone tumors, human osteosarcoma (OS) is characterized by a high mortality rate in children and adolescents (4). Specifically, approximately 5% of all childhood malignancies and approximately 9% of tumor-related deaths depend on OS (5). This disease often affects the distal femur, proximal tibia, and humerus (6). The prognosis of OS patients is uncertain even when they are subjected to a combination of treatments, including chemotherapy and amputation (5). Despite significant advances in treatment strategies over the past few decades, the overall survival rates of OS patients remain low, especially for metastatic OS (7). Therefore, it is crucial to identify novel biomarkers and ensure effective treatment for patients with OS.

The most common internal chemical modification of mRNA is N6-adenosine methylation, a post-transcriptional modification of RNA yielding N6-methyladenosine (m^6^A) (8). m^6^A network components are distinguished into three subtypes: writers, readers, and erasers (9). Writers facilitated the N6-Adenosine methylation and erasers removed; moreover, m^6^A recognition involves specific reader proteins (10). These m^6^A-related regulatory factors play crucial roles in many physiological and pathological processes by modulating RNA stability, mRNA translation and splicing, and miRNA processing (11–13). In addition, N6-adenosine methylation contributes to the initiation and development of several cancers, namely, breast cancer, liver cancer, colorectal cancer, glioma, hepatoblastoma, and cervical cancer (14–19). Nevertheless, the role and clinical value of m^6^A-related regulatory factors remain unclear in OS.

m^6^A-Methylated lncRNAs regulate a number of pathological and biological processes. For example, by inducing mRNA splicing to mediate immunotherapy and regulate gene expression, m^6^A residues prolong patient survival and affect lactate levels in the TME (20). Additionally, overexpression of *METTL14* significantly inhibited the proliferation, invasion, migration, and apoptosis of OS cells, whereas knockdown of *METTL14* exerted the opposite effects (21). Nevertheless, the causes of abnormal expression and methylation of lncRNAs in OS are unknown. Thus, it is vital to construct transcriptome maps of lncRNA expression profiles and m^6^A modification profiles to understand how these processes affect the prognosis of OC.

Patients with OS can benefit from immune cell score analysis. Indeed, immune risk scores aid in assessing prognosis, facilitate adjuvant treatment of OS, and help explain the link between chemotherapy and immune response, thereby demonstrating the importance of immunotherapy (22). As previously reported in solid tumors, treatment outcomes can be influenced by the extent of immune cell infiltration. According to the type of immune cells that are present in the TME, cancers can be distinguished into tumors that are immunologically active or inflammatory (hot) and tumors that are inactive or non-inflammatory (cold). Cold tumors are associated with poor prognosis because they are insensitive to chemotherapy and immunotherapy. A characteristic feature of febrile neoplasms is the infiltration of leukocytes, including CD8^+^ T cells (23). The immune score is a cytotoxic immune response index that can improve the prognosis of collision tumors and guide therapeutic strategies (24). However, few studies have examined the relationship between m^6^A-associated lncRNAs and immune cell infiltration in OS. Therefore, it is necessary to study immune cell infiltration within the TME to clarify the relationship between the latter and OS clinicopathological parameters.

This study focused on the epigenetic mechanisms by which m^6^A-related lncRNAs mediate the development and differentiation of immune cells. We first evaluated the prognostic value and expression status of m^6^A-related lncRNAs by analyzing public genome databases of OS patients. Additionally, we developed clustering subtypes based on the expression levels of prognostic m^6^A-related lncRNAs to determine their relationships with N6-adenosine methylation, TME scores, target gene expression, prognosis, and infiltration of immune cells. Pathway and functional gene set enrichment analyses (GSEA) allowed to further pinpoint the pathways involved in the process. Finally, Lasso regression was used to test the constructed m^6^A-related lncRNA prognostic model.

## Materials and Methods

### Sample Data Acquisition and Collation

The TARGET-OS gene expression (RNA-Seq) dataset (https://ocg.cancer.gov/programs/target) provided clinical data of 88 patients with OS. The GTEx datasets were obtained from the Genotype-Tissue Expression database (http://commonfund.nih.gov/GTEx). Using PERL software (https://www.perl.org/), we created an mRNA matrix and processed transcriptomic data with the corresponding script for gene ID transformation, which enabled to process the clinical data. The software was installed in a path named in English with no spaces.

### Identification of m^6^A-Related lncRNAs

To identify mRNAs and lncRNAs, we developed a human configuration file and a gene expression matrix using PERL software, including the expression levels of m^6^A-related lncRNAs derived from the collated transcriptomic data. After running the script file, we were able to determine the nature of the genes and also the expression levels of mRNAs and lncRNAs. Gene IDs were converted into gene names using the Ensembl database (http://asia.ensembl.org/info/data/index.html). Using the limma package (http://bioconductor.riken.jp/packages/3.0/bioc/html/limma.html) in R software (https://www.r-project.org/), we extracted data on m^6^A-related gene expression, and also the corresponding gene names, based on gene types (i.e., readers, writers, and erasers). We then used co-expression analysis to examine the relationship between the expression of m^6^A-related genes and that of lncRNAs. The resulting correlation coefficients indicated regulatory hypotaxis between these elements. Using the limma package, we obtained expression data of m^6^A-related lncRNAs. Correlations were visualized in a network plot generated using the igraph package. Using the limma package, clinical survival data were cross-referenced with m^6^A-related lncRNA expression data. This analysis revealed lncRNAs related to prognosis, which were selected and utilized to calculate hazard ratios and confidence intervals using the survival package. A forest plot was generated to visualize univariate Cox regression coefficients. Finally, we computed and analyzed differences in m^6^A-related lncRNA expression between tumor tissues and normal tissues using the reshape2, pheatmap, limma, and ggpubr packages in R software. Differences were considered statistically significant at P <0.05. Heatmaps and boxplots were constructed to visualize differences in gene expression.

### Exploring the Role and Function of m^6^A-Related lncRNAs

Based on lncRNA expression data (https://bioconductor.org/install/), we classified m^6^A-associated lncRNAs that were related to prognosis into clusters 1 and 2 using the Consensus Cluster-Plus and limma packages. In our calculations, the following parameters were used: clusterAlg = “km” and clusterNum = “2”. Furthermore, we used the survminer and survival packages to assess the prognostic value of m^6^A-related lncRNAs, and the pheatmap package to generate a heatmap enabling to identify differentially expressed lncRNAs related to prognosis in each cluster, and analyze their correlation with clinicopathological parameters. Using the limma package, we identified different types of tissues and differential target gene expression in the related OS subtypes. The common names of the target genes were obtained from the NCBI (https://www.ncbi.nlm.nih.gov). Finally, using the limma package, we conducted correlation analyses to clarify the relationship between the expression of prognostic m^6^A-associated lncRNAs and that of target genes in OS. Differences were considered statistically significant at P <0.05.

### Analysis of the Tumor Microenvironment and Immune Cell Infiltration

We applied the preprocessor, limma, and e1071 packages to explore the levels of immune cell infiltration in the samples and calculated the number of infiltrating immune cells. We analyzed the TME using the limma package, and calculated its immune score, ESTIMATE score, and stromal score. These scores predicted tumor purity. Moreover, we estimated differences in the abundance of various immune cell subtypes between the clusters using the limma package, and visualized them using the vioplot package. The degree of infiltration of each immune cell type was also analyzed using the limma package, and the results were plotted on a boxplot. In parallel, we used the limma package to perform differential analysis of the TME based on the immune score, ESTIMATE score, and stromal score, and produced boxplots to determine the tumor cell purity of different OS subtypes. Next, gene set enrichment analysis (GSEA) was performed (http://www.gsea-msigdb.org/gsea/index.jsp) to identify pathways and related functions showing differential enrichment among samples, and PERL software was used to analyze the results. To understand whether the gene set enrichment of different clusters was dynamic or static, using the calculated scores and the generated plots, we classified each sample as “H” or “L” based on its assignation to a high- or low-risk lncRNA cluster, respectively. This analysis was performed by setting the ordering mode of the gene list as “descending”, the ranking gene metric as “Signal2Noise”, the sorting mode of the gene list as “real”, the normalization model as “meandiv”, and the permutation type and number to “no collapse” and “phenotype”, and “1000”, respectively (25). FDR <0.05 was set as threshold for statistical significance.

### Prognostic Value of m^6^A−Related lncRNAs

We performed Lasso regression to generate a prognostic model. Considering the median risk score of prognostic m^6^A-associated lncRNAs, the samples were categorized as high- or low-risk. Lasso regression was carried out using 50% training samples and 50% test samples, and the corresponding plots were obtained. We then compared the high- and low-risk survival curves. To assess the accuracy of our model in predicting the survival rate of patients with OS, we generated a receiver operating characteristic (ROC) curve using the time ROC package. Data on the risk score, the risk associated with m^6^A-related lncRNAs, and survival status were combined to create a risk curve. Through independent prognostic factor analysis, we evaluated the independence of our model from other prognostic clinical factors. Multivariate and univariate analyses were performed, and the hazard ratios were calculated. We then validated our model using clinical data from various sources to assess its applicability to different clinical contexts, and conducted correlation analyses of clinical characteristics and risk scores to assess the relationship between these factors and m^6^A-associated lncRNAs. Using the limma and pheatmap packages, we generated a heatmap. Boxplots were constructed to assess the relationship between clinical data and risk scores. Using differential gene expression analysis, we evaluated differences in target gene expression between the risk groups, performed correlation analyses to determine the relationship between risk scores and levels of infiltrating immune cells, and visualized this nexus on a scatter plot to determine the beneficial role of immune cells.

### Immunofluorescence

Primary antibodies against *ZBTB32*, *DEPTOR*, and *SPAG4* were purchased from Abcam, Cell Signaling Technology, and Santa Cruz Biotechnology, respectively. Tissue samples embedded in paraffin were prepared for sectioning and antigen retrieval. According to the standard procedure, slides were incubated with primary antibodies overnight at 4°C. The slides were then washed three times with PBS, and incubated with secondary antibody at room temperature for 50 min in the dark. Subsequently, DAPI was used to stain nuclei. Finally, images were captured using a fluorescence microscope.

## Results

### Identification of m^6^A-Related lncRNAs

**Figure 1:**
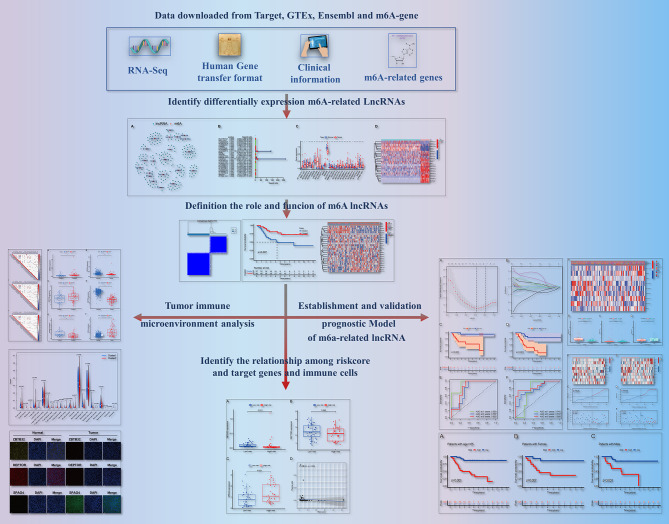
Flow diagram of this study.

**Figure 2:**
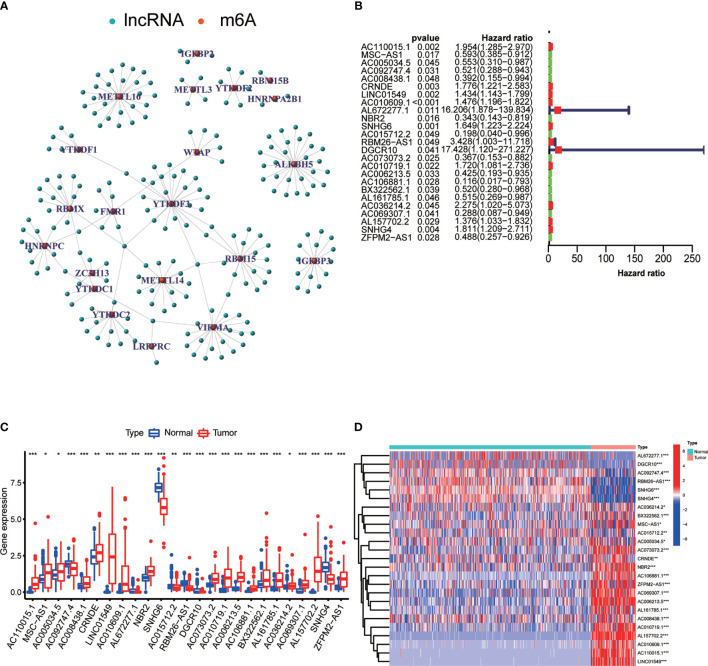
Differential expression of m^6^A-associated lncRNAs in relation to the prognosis of OS. **(A)** Network plot showing the relationship between the expression of lncRNAs and that of m^6^A-associated genes. **(B)** Forest plot showing the results of Cox regression analysis using univariate data. We extracted lncRNA-associated prognostic data and calculated hazard ratios and confidence intervals. Red indicates high risk, while green indicates low risk. **(C)** Boxplot showing differences in the expression levels of m^6^A-related lncRNAs associated with the prognosis of OS between normal tissues and tumor tissues. ***P < 0.001; **P < 0.01; *P < 0.05. **(D)** Heatmap showing differential expression of m^6^A-related lncRNAs in tumor tissues and normal tissues. ***P < 0.001; **P < 0.01; *P < 0.05. Red indicates high expression levels, while blue indicates low expression levels. Samples are reported on the abscissa and lncRNAs with prognostic value are reported on the ordinate.

As shown in **Figure 1**, we created a detailed workflow for our entire study. First, the expression data of m^6^A-related genes were derived from the collated transcriptomic data by discriminating mRNAs from lncRNAs. Co-expression analysis showed a correlation between the expression of lncRNAs and that of m^6^A-related genes. To visualize this correlation, we generated a network plot (**Figure 2A**). Also, a forest plot was generated to visualize the results of univariate Cox regression analysis (**Figure 2B**). m^6^A-Associated lncRNAs were deemed to be prognostic at P <0.05, and *DGCR5 (DCGR10)* presented the highest hazard ratio. The heatmap and box plot in **Figures 2C, D** show differences in the expression levels of prognostic m^6^A-related lncRNAs between normal and tumor tissues. Among these, a total of 25 lncRNAs displayed differential expression between tumor tissues and normal tissues. The expression of some of these lncRNAs was lower in tumors, whereas that of others was higher (P <0.05).

### The Role of m^6^A-Associated lncRNAs

**Figure 3:**
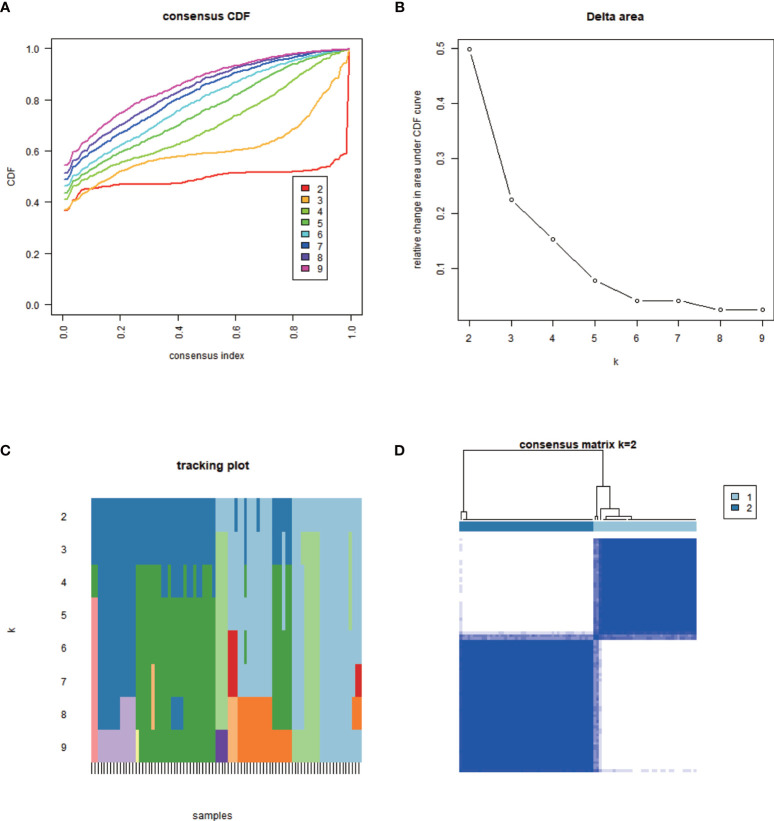
Classification of m^6^A-associated lncRNAs with prognostic value. The minimum overlap based on expression levels occurred at K = 2, together with the lowest cumulative distribution function (CDF) value; therefore, lncRNAs were classified into two clusters: cluster 1 and cluster 2. **(A)** Empirical CDF graph for K = 2–9. **(B)** Relative changes in the area under the CDF curve for K = 2–9. **(C)** Trace plot providing an overview of projected cluster members for different K values and of the cluster history relative to earlier clusters. **(D)** Consensus clustering matrix at K = 2.

**Figure 4:**
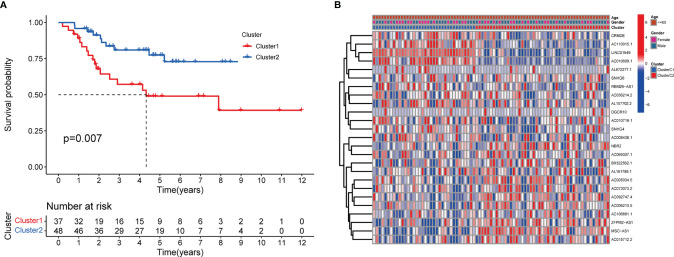
Survival analysis and correlation of m^6^A-associated lncRNAs with clinical parameters. **(A)** Survival analysis revealed that, between the lncRNA subtypes, cluster 2 was associated with a higher 5-year survival rate (P = 0.007). **(B)** Heatmap showing differential expression of lncRNAs associated with prognosis and their correlation with clinicopathological parameters in the different clusters. Red indicates high expression levels, while blue indicates low expression levels. LncRNAs related to prognosis are reported on the ordinate and samples are reported on the abscissa.

**Figure 5:**
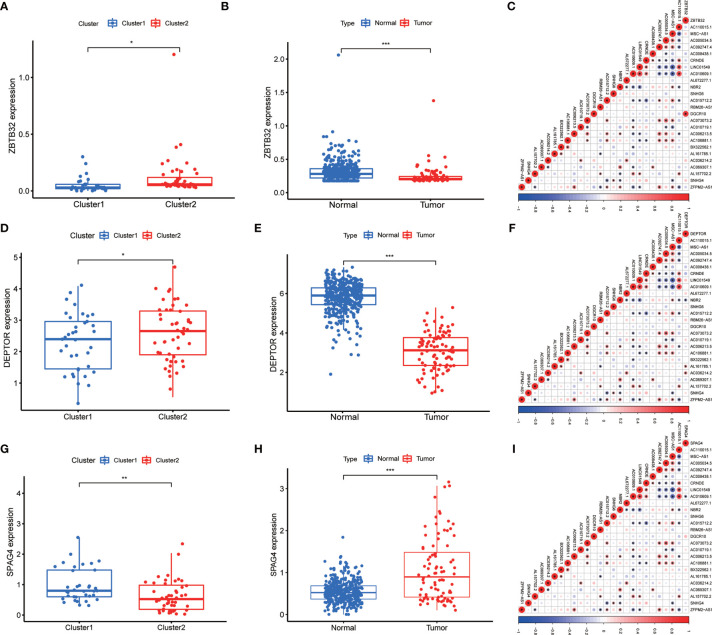
Differential expression of target genes in different OS subtypes, and correlation analysis between m^6^A-associated lncRNA levels and target gene expression. Differences in the expression of *ZBTB32*, *DEPTOR*, and *SPAG4* in various subtypes **(A, D, G)** and different tissue types **(B, E, H)**. Correlation analysis of the relationship between the expression of the target genes *ZBTB32*, *DEPTOR*, and *SPAG4* and that of prognostic m^6^A-related lncRNAs in OS tissues **(C, F, I)**. Red indicates positive relationships, while blue indicates negative relationships. *P < 0.05, **P < 0.01, ***P < 0.001.

The minimum overlap and lowest cumulative distribution function (CDF) value, in terms of lncRNA expression, occurred at K = 2. Therefore, lncRNAs were grouped into two groups (**Figure 3** and **Figures S1**, **S2**), named clusters 1 and 2. The prognostic value of m^6^A-related lncRNAs was assessed based on survival analysis stratified by lncRNA subtype; this analysis revealed that lncRNAs from cluster 1 were associated with a lower survival rate than that of lncRNAs from cluster 2 (**Figure 4**; P = 0.007). Moreover, differences in the expression of three target plasma cell genes between the different types of tissue specimens, and also among the related subtypes, were investigated (**Figure 5**). The expression levels of *ZBTB32* and *DEPTOR* were higher in normal tissues than in tumor tissues (**Figure 5B, E**; P <0.001), while the opposite was true for *SPAG4* expression (**Figure 5F**, P <0.001). In addition, we performed gene correlation analysis to determine the relationship between OS-prognostic m^6^A-associated lncRNAs and target genes; this showed a correlation between the expression of *DEPTOR* and that of the prognostic m^6^A-related lncRNA *AC036214.2*; and between the expression of *ZBTB32* and *SPAG4* and that of the prognostic m^6^A-related lncRNA *DGCR5 (DCGR10)* (**Figures 5C, F, I**; P <0.05).

### Correlation Between the Expression of *ZBTB32*, *DEPTOR*, and *SPAG4* and That of Prognostic m^6^A-Related lncRNAs in OS

**Figure 6:**
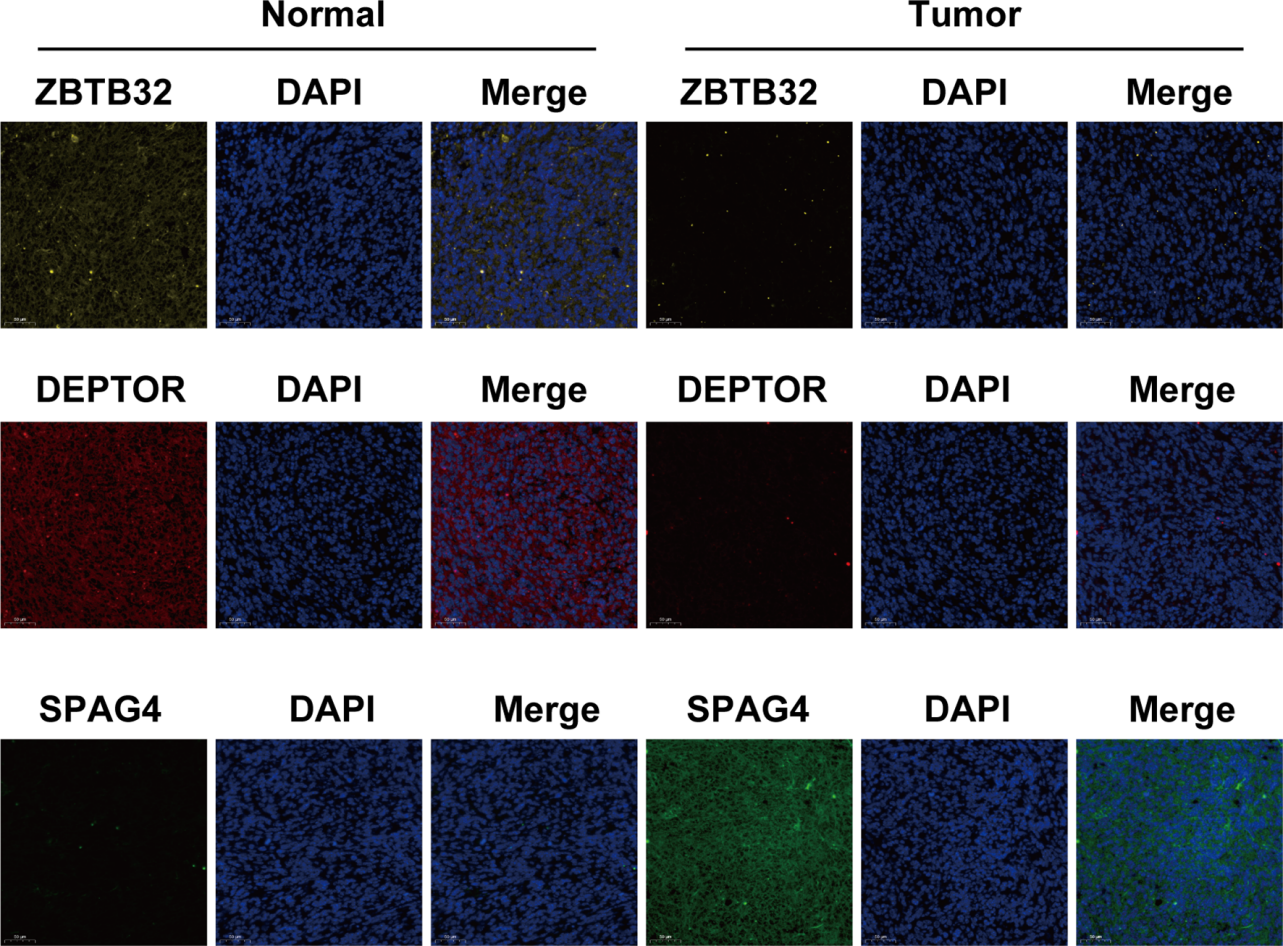
The expression of *ZBTB32* and *DEPTOR* decreased while that of *SPAG4* increased in OS tissues, as revealed by immunofluorescence staining of *ZBTB32*, *DEPTOR*, and *SPAG4* in OS tissues and paired normal bone tissues.

Immunofluorescence assays were carried out to verify the role of the three above-mentioned genes in OS (**Figure 6**). Compared to those of normal bone tissue (left), *ZBTB32* (upper panel) and *DEPTOR* (middle panel) protein levels were consistently lower in OS tissues (right), while *SPAG4* (bottom panel) protein levels were higher.

### Role of the TME and Infiltration of Immune Cells

**Figure 7:**
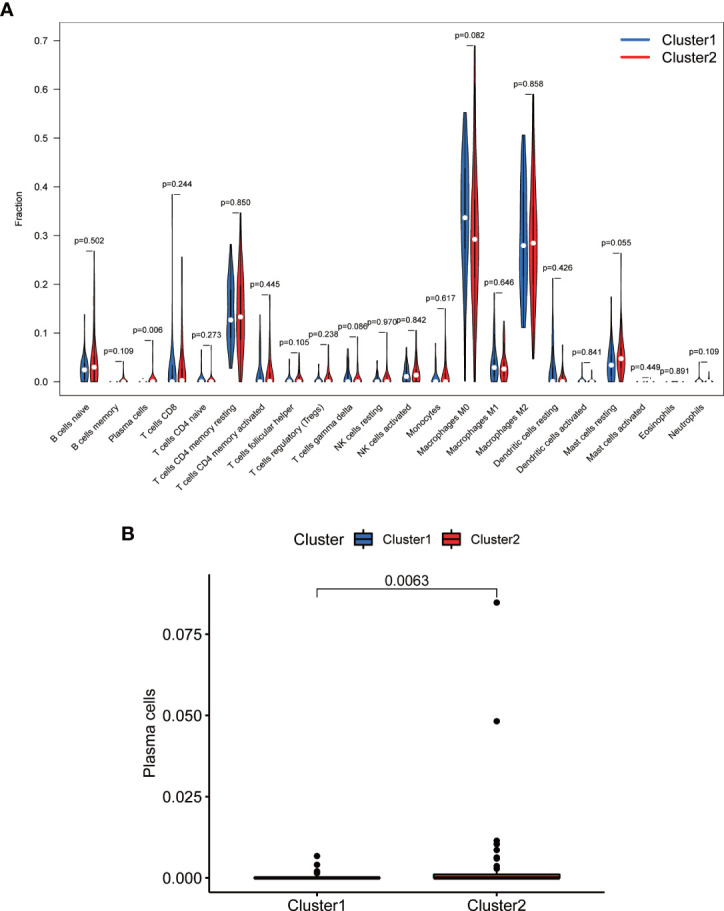
Comparison of immune cell infiltration in different clusters. **(A)** Violin plot. **(B)** Boxplot. Cluster 2 exhibited higher levels of immune cells, in particular of plasma cells (P <0.05).

**Figure 8:**
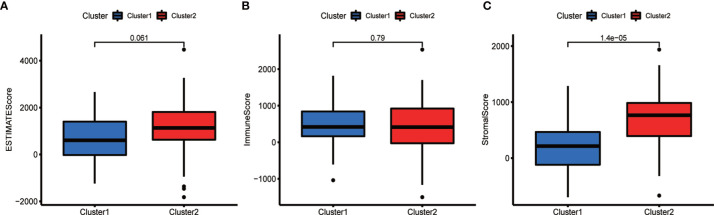
Comparison of the tumor microenvironment (TME) in the two subtypes. Analysis of differences in the TME of different OS subtypes. The ESTIMATE score **(A)** and the immune score **(B)** did not differ significantly between the two types, whereas the stroma score **(C)** was higher in cluster 2 (P <0.05).

**Figure 9:**
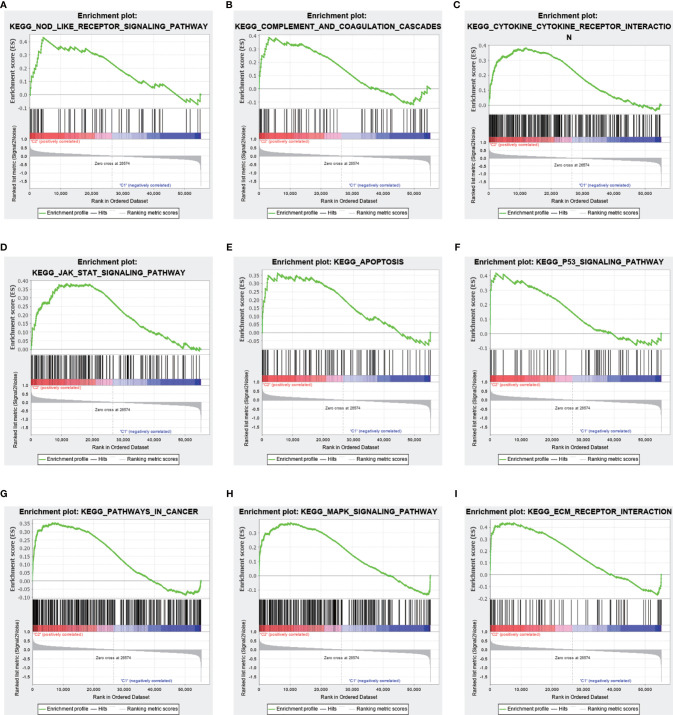
Differences in the enrichment of pathways and related functions between the two clusters based on gene set enrichment analysis (GSEA). Upper panel, partial results of GSEA. **(A–I)** Multiple pathways and functions related to cancer were found to be enriched in low-risk m 6 A-related lncRNAs, belonging to cluster 2. The latter is positively correlated to all gene sets (P <0.05).

TME analysis was performed to examine the degree of immune cell infiltration in the samples. The results of differential infiltration analysis in the two clusters were reported in a violin plot (**Figure 7A**). We also plotted the degree of immune cell infiltration within each OS cluster (**Figure 7B** and **Figures S4**–**S6**). These analyses revealed that cluster 2 exhibited a higher concentration of plasma cells than that of cluster 1 (P <0.05). We then examined the correlation between risk scores and immune cell infiltration levels in the TME of different OS subtypes, and generated boxplots to illustrate tumor cell purity in each of these subtypes (**Figures 8A–C**). Cluster 2 had a significantly higher stromal score than that of cluster 1, indicating a lower purity in the TME (P <0.05). We then conducted GSEA to identify pathways and related functions that showed differential enrichment between the samples (**Figure 9** and **Figure S3**). A partial list of the GSEA results is reported in **Figure 9**. The gene set with the highest enrichment was NOD-like receptor signaling pathway. Cluster 2 was positively associated with all gene sets (P <0.05).

### Prognostic Value of m^6^A-Associated lncRNAs

**Figure 10:**
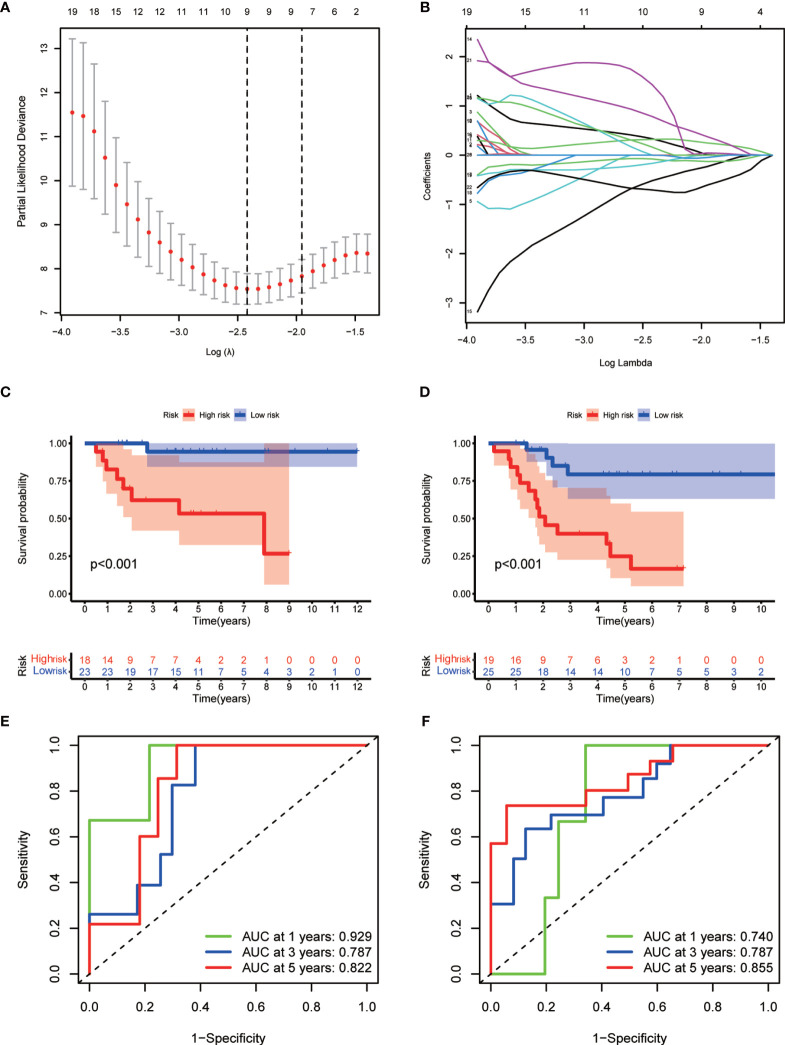
Prognostic model. **(A, B)** Results of Lasso regression for the construction of the prognostic model. **(C, D)** Survival curves related to the test group **(C)** and the training group **(D)** at P <0.05. **(E, F)** Receiver operating characteristic (ROC) curve indicating the accuracy of the model for survival prediction (left, test group; right, training group; area under the curve [AUC] >0.5).

**Figure 11:**
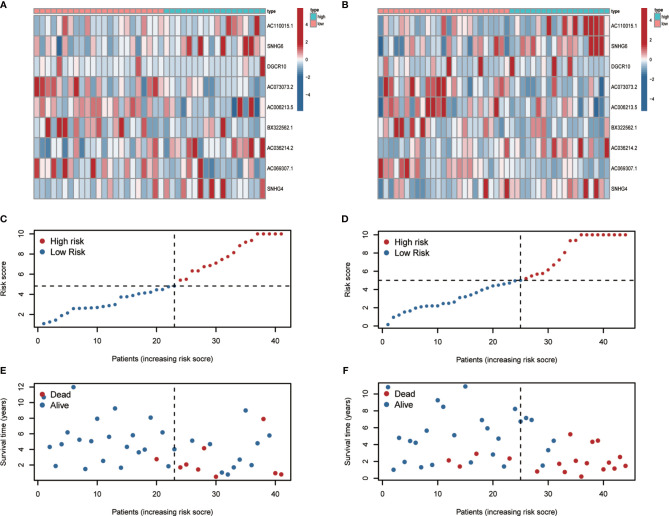
Heatmap, risk-related curve, and spot plot of the test group **(A, C, E)** and the training group **(B, D, F)**. The death rate and high-risk ratio increased with increasing risk scores.

**Figure 12:**
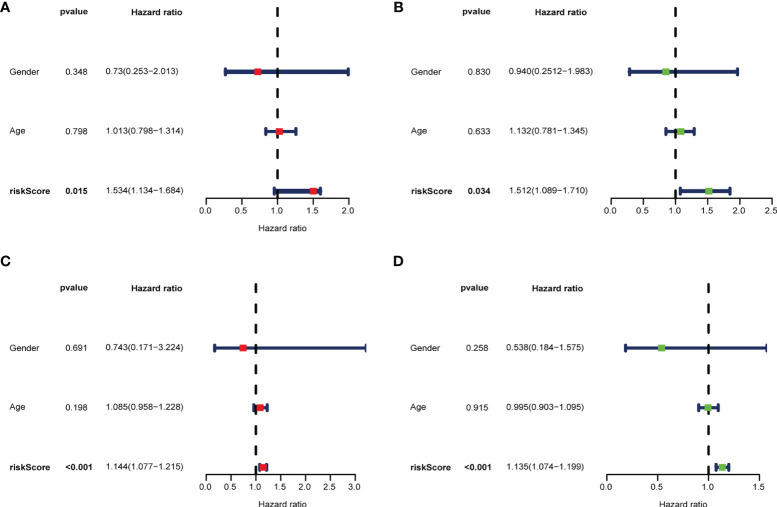
Multivariate **(A, C)** and univariate **(B, D)** independent prognostic factor analyses in the test group **(A, B)** and the training group **(C, D)**. The risk score was found to be an independent risk factor for OC prognosis (P <0.05).

**Figure 13:**
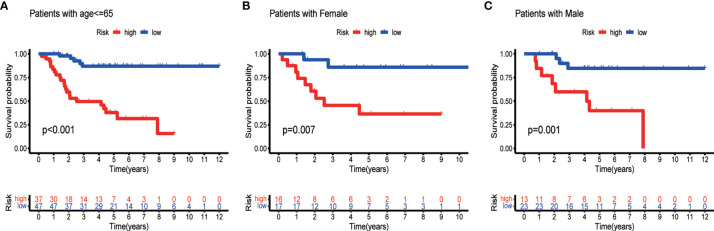
Survival curves for model validation. The model resulted valid for various clinical groups, differing by age **(A)** or gender **(B, C)**, at P <0.05.

**Figure 14:**
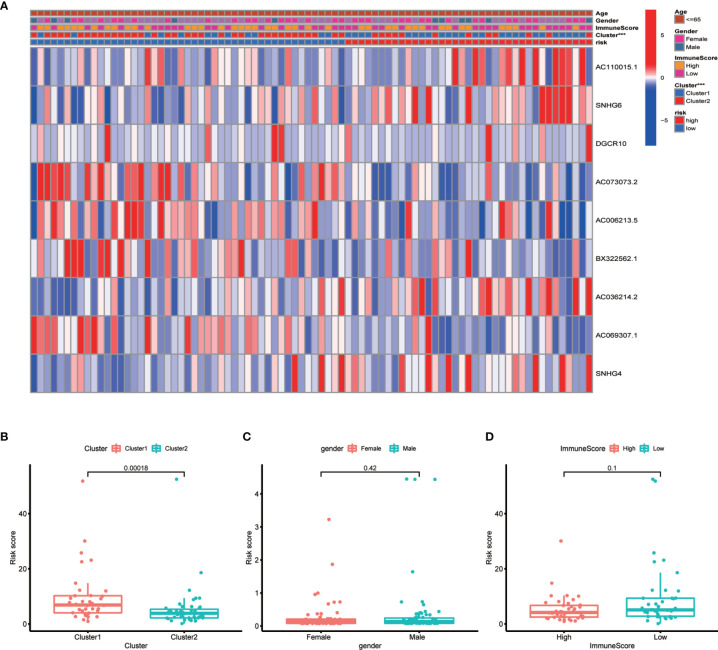
Correlation between risk scores and clinical outcome. **(A)** Heatmap showing the correlation between risk scores and clinical outcome. **(B–D)** Boxplots showing the relationship between risk scores and clusters, gender, and immune scores. Clusters and risk score were closely correlated (P <0.05, ***P < 0.001).

**Figure 15:**
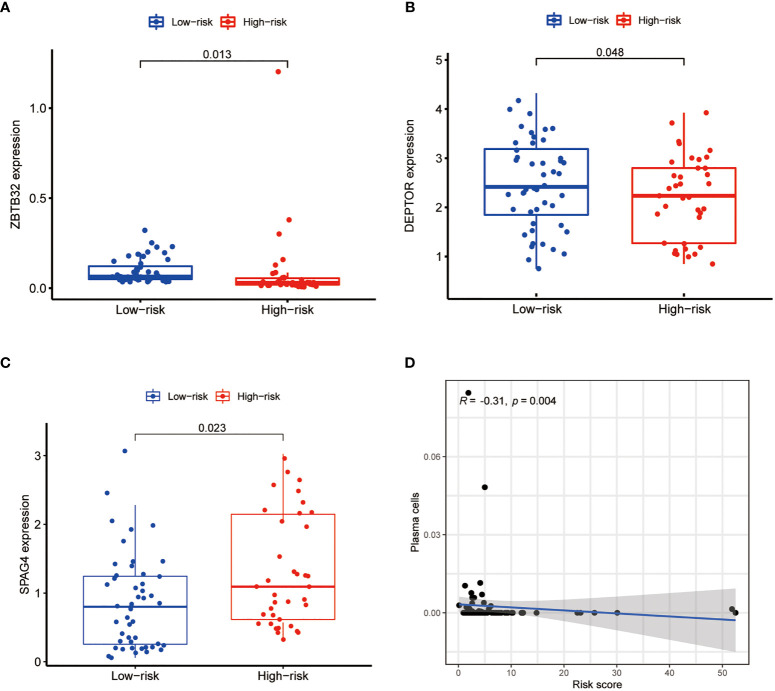
Differential expression analysis of target genes and scatter plots showing the correlation of immune cell levels with risk scores. The expression of *ZBTB32* **(A)** and *DEPTOR* **(B)** was lower in the high-risk group, whereas that of *SPAG4* **(C)** was higher in the high-risk group (P <0.05). **(D)** Plasma cell levels were negatively correlated with the risk score (R <0, P <0.05).

To develop a prognostic model, we carried out Lasso regression and categorized all samples into a high-risk group and a low-risk group based on the median value of the risk score. Lasso regression was performed on a test group (50%) and a training group (50%) (**Figures 10A, B**). Patients were then divided into two subgroups, that is, a high-risk group and a low-risk group, based on their median risk score. Subsequent survival curve analysis revealed that patients exhibiting higher risk scores, from both the test and the training groups, had poorer prognoses. Conversely, patients in the low-risk group showed a higher survival rate than that of patients in the high-risk group (P <0.05; **Figures 10C, D**). Moreover, the analysis of ROC curves showed that a prognostic signature based on m^6^A-associated lncRNAs was a reliable predictor of test subtype [area under the curve (AUC), 0.929, 0.787, and 0.822 for 1, 3, and 5 years, respectively] and training subtype (AUC, 0.740, 0.787, and 0.855 for 1, 3, and 5 years, respectively; **Figures 10E, F**). **Figure 11** illustrates the expression patterns (heatmap, upper panel), risk score (middle panel), and survival time (bottom panel) associated to m^6^A-related lncRNA prognostic regulators. Notably, the death rate and high-risk ratio increased with increasing risk scores. To examine the independence of our model from other clinical prognostic factors affecting patient outcomes, we performed independent prognostic factor analysis (**Figure 12**), which demonstrated that the risk score acts as an independent prognostic factor for OS (P <0.05). As illustrated in **Figure 13**, clinical validation of the model confirmed its suitability under different clinical parameters, including sex and age (P <0.05), suggesting that our model is broadly applicable. Furthermore, correlation analysis of clinical characteristics and risk scores was performed to evaluate how differences in the expression of m^6^A-related lncRNAs between low- and high-risk groups correlated with clinical characteristics (**Figure 14A**), and also with risk prognosis (**Figures 14B–D**). Notably, *AC110015.1*, *SNHG6*, and *AC036214.2* showed high expression in the high-risk group, whereas *AC073073.2*, *AC0086213.5*, and *BX322562.1* were poorly expressed in the low-risk group. Moreover, the clusters were closely related to the risk score (P <0.05). Finally, differences in the expression of target genes between the different risk groups in our OS model were observed (**Figures 15A–C**). In particular, *ZBTB32* and *DEPTOR* expression levels were lower in the high-risk group than in the low-risk group (P <0.001), while the opposite was true for *SPAG4* expression. A scatter plot was constructed to visualize this nexus and assign a beneficial or detrimental role to immune cells (**Figure 15D**). Plasma cells correlated negatively with risk scores (R <0 and P <0.01).

## Discussion

Previous studies have demonstrated that the downregulation or upregulation of N6-adenosine methylation regulators is related to the onset and development of various tumors, such as non-small cell lung cancer, acute myeloid leukemia, glioblastoma, breast cancer, and liver cancer (14, 26–30). Moreover, the same methylation regulators may function differently in different tumor tissues. Epigenetic modifications of proteins or DNA are crucial for cancer progression and prognosis (31, 32). Previous studies have focused primarily on m^6^A-related genes and pathways associated with cancer metastasis and prognosis; however, m^6^A-related lncRNAs have not been systematically analyzed in OS. Thus, the identification and analysis of lncRNAs associated with m^6^A are of considerable importance, as they can guide future OS research. Additionally, in this study N6-adenosine methylation of RNA molecules was also found to correlate with prognosis and levels of immune cell infiltration in the TME of OS tissues.

In the current study, we identified mRNAs and lncRNAs associated with m^6^A gene expression. Furthermore, co-expression analysis was performed to determine whether the expression of m^6^A-related genes was correlated with lncRNA expression. The resulting co-expression network plot revealed that the expression of several lncRNAs is correlated with that of m^6^A-related genes in OS. This finding sparked our interest in m^6^A-related lncRNAs and their role in OS; therefore, we identified lncRNAs associated with the prognosis of OS, calculated their hazard ratios and confidence intervals, and observed a close correlation between the expression of m^6^A-related lncRNAs and prognosis of OS, as revealed by Cox regression analysis of univariate data. Cancer development and m^6^A modifications are closely linked. Indeed, N6-adenosine methylation influences lncRNA splicing, thereby possibly altering cancer progression (33, 34). In our study, we found 25 lncRNAs associated with the prognosis of OS and m^6^A modifications, whose expression varied between tumor and normal tissues. A number of these lncRNAs were highly expressed in tumor tissues, while others were highly expressed in normal tissues (P <0.05).

Interestingly, *PVT1*, a gene significantly associated with OS survival, is upregulated in cancers through *ALKBH5*-mediated N6-adenosine methylation (35, 36). Recent studies have suggested that *PVT1* may promote glycolysis, metastasis, and doxorubicin (DXR) resistance in OS cells (37, 38). LncRNA modifications can have a profound effect on the protein interaction network and can thus affect cancer progression (39). Altogether, the above findings revealed that a number of m^6^A-associated lncRNAs are highly expressed in tumor tissues, while others are highly expressed in normal tissues. Additionally, m^6^A-related lncRNAs can function as either tumor suppressors or oncogenes.

The role of m^6^A-associated lncRNAs was further analyzed. To evaluate the prognostic value of m^6^A-related lncRNAs, survival analysis based on lncRNA expression in different OS subtypes was conducted. The expression of lncRNAs associated with the low-risk group was found to be beneficial for the prognosis of OS. Furthermore, lncRNA expression was associated with OS survival rates. Similar results were reported by Chen et al., who demonstrated that the m^6^A demethylase *ALKBH5* inhibits the binding of the reader protein *YTHDF2* to the lncRNA *PVT1*, thereby suppressing its degradation (36). Notably, *PVT1*, whose expression is mediated by m^6^A, serves as a valuable prognostic predictor for patients with OS. Interestingly, m^6^A-related lncRNAs were expressed at similar levels in all clusters, possibly because of their low expression levels in OS. In addition, the study of the role of modified lncRNAs in OS progression is still limited. Therefore, further research on N6-adenosine methylation of lncRNAs and m^6^A recognition is urgently needed to validate our findings.

*ZBTB32* (zinc finger and BTB domain containing 32), a gene located on chromosome 19, is widely expressed in the testis, lymph nodes, and other tissues. By targeting the *Zpo2* to *GATA3* promoters, *ZBTB32* promotes the deregulation of *GATA3* target genes, leading to the progression of aggressive breast cancer (40). For instance, human activated B cells in diffuse large B-cell lymphoma (DLBCL) carry *ZBTB32* as the most consistently expressed gene (41). Conversely, *CIITA*, encoding an MHC class II transactivator, is silenced during the differentiation of B cells into plasma cells (42). However, *ZBTB32* was identified as an early repressor of *CIITA* in an *ex vivo* plasma cell differentiation model. Therefore, *ZBTB32* expression seems to primarily affect the maintenance of terminal plasma cells (43). In our study, *ZBTB32* was found to be downregulated in OS samples, pointing at this gene as a potential tumor suppressor. However, higher expression of *ZBTB32* was observed in cluster 2, possibly resulting in better prognosis of OC. Moreover, co-expression analysis of target genes revealed that *ZBTB32* expression was significantly correlated with that of m^6^A-associated lncRNAs in OS tissues. In particular, *ZBTB32* expression was highly correlated with *DGCR5 (DCGR10)* expression. As shown in **Figure 2B**, *DGCR5 (DCGR10)* expression was decreased in tissues from OS patients, resulting in decreased expression of *ZBTB32*. This observation further supports our hypothesis that *DGCR5 (DCGR10)* acts as a tumor suppressor.

In mature myeloma cells, *DEPTOR* (DEP domain containing MTOR interacting protein) increases sensitivity to therapeutic agents by inhibiting the activity of mTOR kinases (44, 45). Similarly, through the inhibition of *mTORC1/2* signals, *DEPTOR* serves as a tumor suppressor in human prostate cancer (46, 47). In this study we observed low *DEPTOR* levels in OS patients, indicating that this gene might function as an oncosuppressor. In addition, *DEPTOR* expression was higher in cluster 2, suggesting its role in ensuring a favorable prognosis for OS. The results of our study concur with those of Hu et al., who found that downregulation of *DEPTOR* reduces the survival of OS patients *via* altered regulation of the *PI3K/Akt/mTOR* pathway (48). Correlation analysis between the expression of target genes and that of prognostic m^6^A-associated lncRNAs in OS have revealed that *DEPTOR* expression is closely associated with that of several m^6^A-related lncRNAs. Additionally, *DEPTOR* expression was negatively correlated with that of *NBR2* and positively correlated with that of *AC036214.2* and *AL161785.1*. Thus, these genes may serve as therapeutic targets for OS treatment.

*SPAG4* (sperm associated antigen 4), a SUN family protein, was first discovered in the mammalian sperm tail. This protein interacts with outer dense fiber of sperm tails 1 (ODF1) and plays a crucial role in spermatogenesis and sperm motility (49). *SPAG4* has been reported as a clinically relevant cancer marker (50). Recently, *SPAG4* knockdown has been shown to reduce the invasion and migration abilities of cancer cells, while its overexpression enhanced these abilities of tumor cells (51, 52). In the present study, we found that the expression of *SPAG4* was higher in patients with OS, suggesting that this gene might act as a tumor promoter. In addition, *SPAG4* expression was low in cluster 2, suggesting that it may be detrimental to OS survival. Correlation analysis between the expression of *SPAG4* and that of prognostic m^6^A-associated lncRNAs in OS revealed that *SPAG4* expression was most negatively correlated with *AC010609.1* expression.

Additionally, we measured the levels of infiltrating immune cells in order to determine the significance of immune cell infiltration in the TME of OS. Differential immune cell infiltration analysis revealed that cluster 2 was enriched in immune plasma cells. As previously stated, cluster 2 represents lower-risk OS cases characterized by good prognosis. Therefore, plasma cells infiltrating the TME may benefit the prognosis of the patient. This conclusion is consistent with the findings of Weiner et al., who showed that plasma cell enrichment correlated with prognosis and a fully functional regulation of immunity in human prostate cancer (53). Furthermore, it is becoming increasingly evident that plasma cells and intratumoral antibody production can significantly impact the antitumor response. In particular, various studies have shown that plasma cells are very strongly associated with long-term survival in many cancers (54–57). In this study, we also performed differential analysis of TME for various subtypes of cancer to examine the purity of tumor cells within different clusters. Notably, the scores of cluster 2 were consistently higher than those of cluster 1, suggesting a lower tumor cell purity and a greater number of immune and stromal cells within the TME. These results support the hypothesis that OS tissues belonging to cluster 2 are characterized by low risk scores and good prognosis. Similarly, multiple studies have demonstrated that the levels of tumor-infiltrating immune cells are closely associated with the prognosis of OS and are reliable predictors of OS outcome (22, 58–60). Altogether, our findings and previous studies confirm our hypothesis that immune cell infiltration affects patient prognosis. In particular, higher stromal scores result in less pure tumors and better prognosis than lower immune scores.

Recently, N6-adenosine methylation has been found to play a critical role in the tumor immune microenvironment and to be associated with cancer prognosis (61–65). In fact, N6-adenosine methylation regulates not only the development or function of immune cells and other stromal cells within the TME, but also the responses of the TME to various stimuli, such as hypoxia, internal or external cellular stress, metabolic dysregulation, chronic inflammation, and *TGF-β* (66, 67). Meanwhile, the TME also plays a key role in the complex regulatory network of N6-adenosine methylation, thereby affecting tumor initiation, progression, and response to therapy (67). Nevertheless, such regulatory relationship between the TME and N6-adenosine methylation and its role in tumor pathology require further study.

Next, we conducted GSEA. The most significantly enriched gene set was the NOD-like receptor signaling pathway. The function of this signaling pathway in OS is relatively unknown, and may thus be explored in future research to further improve the diagnosis and treatment of OS. We speculate that the expression of m^6^A-related lncRNAs may impact OS antitumor immunity by modulating the *NOD*-like receptor signaling pathway. Finally, we established a prognostic model based on m^6^A-related lncRNAs using Lasso regression. In both the test and the training group, the low-risk subtype displayed a higher survival rate than that of the high-risk subtype. Therefore, m^6^A-related lncRNA expression allows to accurately predict the outcome of patients with OS. In addition, this model for predicting disease survival has considerable accuracy. In fact, the number of deaths and the proportion of high-risk individuals increased as the risk score increased. Moreover, the model was not affected by other clinical prognostic factors that may affect the outcome of OS, and was found to be applicable to a variety of clinical groups. N6-adenosine methylation of lncRNAs may modulate their interactions with proteins and modify their structure, thereby repressing gene expression (60, 68). Moreover, such modification of lncRNAs may change the subcellular distribution of these molecules, thereby altering their stability and promoting metastasis and tumorigenesis (68).

## Conclusion

In summary, data from the literature and the present study indicate that m^6^A-associated lncRNAs function as epigenetic regulators of plasma cell development. Meanwhile, the expression of m^6^A-related lncRNAs can serve as a clinical indicator for OC prognosis. In the developed OS prognostic model, differential gene expression analysis showed a higher expression of *SPAG4* in the high-risk group, supporting the hypothesis that *SPAG4* acts as a tumor promoter in OS. Although this function of *SPAG4* has been shown in other cancers, only few studies have investigated the role of *SPAG4* in OS cells; therefore, further studies are necessary to confirm these findings. Furthermore, the three examined target genes were found to regulate plasma cell differentiation. Correlation analysis between risk scores and levels of immune cells revealed a negative correlation between the risk score and plasma cell levels. Similarly, Weiner et al. proposed that the presence of plasma cells may improve tumor outcomes and thus represents a positive factor during cancer therapy (53). This hypothesis is consistent with our results of immune cell infiltration analysis stratified by cluster. In conclusion, our results suggest that epigenetic regulation of the development and differentiation of immune cells by m^6^A-associated lncRNAs yields promising predictive markers, and may provide valuable insights into OS treatment by guiding effective immunotherapy.

## Data Availability Statement

The datasets presented in this study can be found in online repositories. The names of the repository/repositories and accession number(s) can be found in the article/**Supplementary Material**.

## Ethics Statement

The studies involving human participants were reviewed and approved by The Third Xiangya Hospital. The patients/participants provided their written informed consent to participate in this study.

## Author Contributions

ZW and XZ: Acquisition and analysis of data. YD, JW, and XW: Conceptual design. ZW, XZ, and XW: Wrote the manuscript. DC and ZL: Data analysis. All authors contributed to the article and approved the submitted version.

## Funding

This work was supported by The Natural Science Foundation of China (81472058); Hunan Science and Technology Innovation Plan (2018SK2105; 422000008); The Fundamental Research Funds for the Central Universities of Central South University (under Grant No. 2020zzts896); Qinghai Key Laboratory of Laboratory Medicine, Qingke Fazheng [2019] No. 105.

## Conflict of Interest

The authors declare that the research was conducted in the absence of any commercial or financial relationships that could be construed as a potential conflict of interest.

## Publisher’s Note

All claims expressed in this article are solely those of the authors and do not necessarily represent those of their affiliated organizations, or those of the publisher, the editors and the reviewers. Any product that may be evaluated in this article, or claim that may be made by its manufacturer, is not guaranteed or endorsed by the publisher.

## Abbreviations

OS, Osteosarcoma; lncRNA, Long non-coding RNA; m^6^A, N6-methyladenosine; TME, Tumor microenvironment; GSEA, Gene set enrichment analysis; ROC, receiver operating characteristic; AUC, area under the curve; *DEPTOR*, DEP domain containing MTOR interacting protein; *ZBTB32*, zinc finger and BTB domain containing 32; *SPAG4*, sperm associated antigen 4.
