# Supplementary figures and images for: N6-Methyladenosine-Related LncRNAs Are Potential Remodeling Indicators in the Tumor Microenvironment and Prognostic Markers in Osteosarcoma
Source: Front Immunol. 2022 Jan 12;12:806189. doi: 10.3389/fimmu.2021.806189 (PMC8790065; doi:10.3389/fimmu.2021.806189)

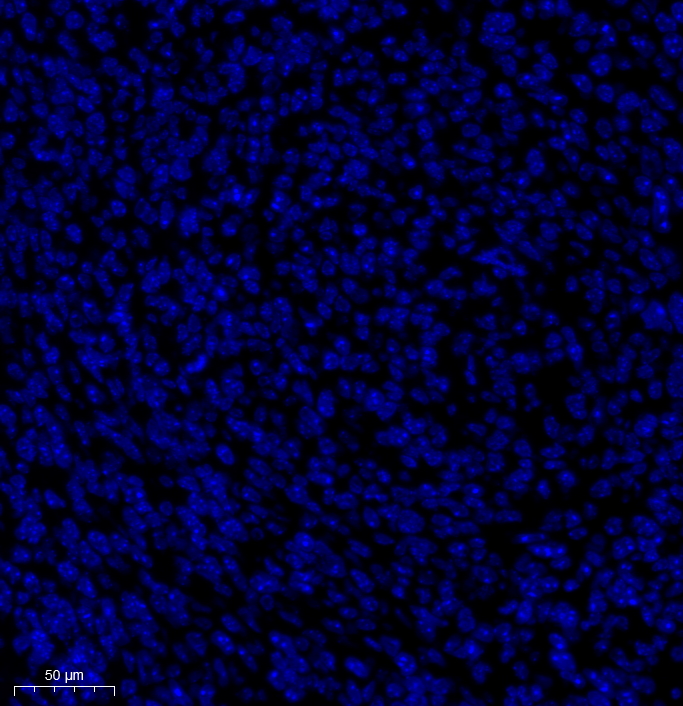

Supplement: Supplementary file 1 [file DataSheet_1.zip › rawdata-IF/DEPTOR/Nc/NC-DAPI.jpg]

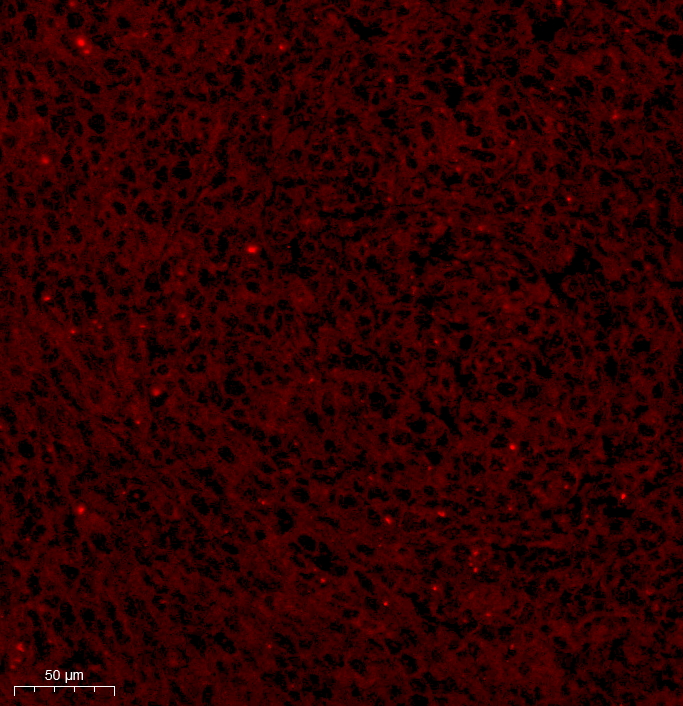

Supplement: Supplementary file 1 [file DataSheet_1.zip › rawdata-IF/DEPTOR/Nc/NC-DEPTOR.jpg]

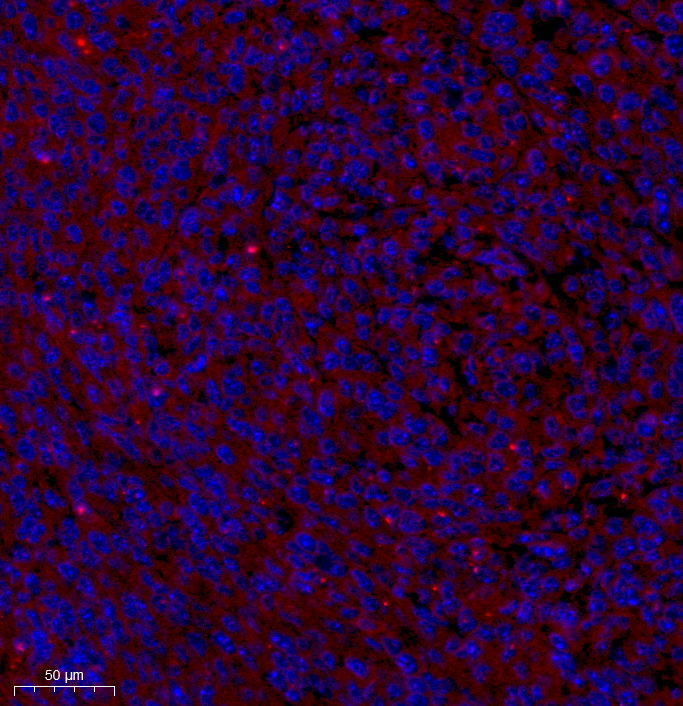

Supplement: Supplementary file 1 [file DataSheet_1.zip › rawdata-IF/DEPTOR/Nc/NC-merge.jpg]

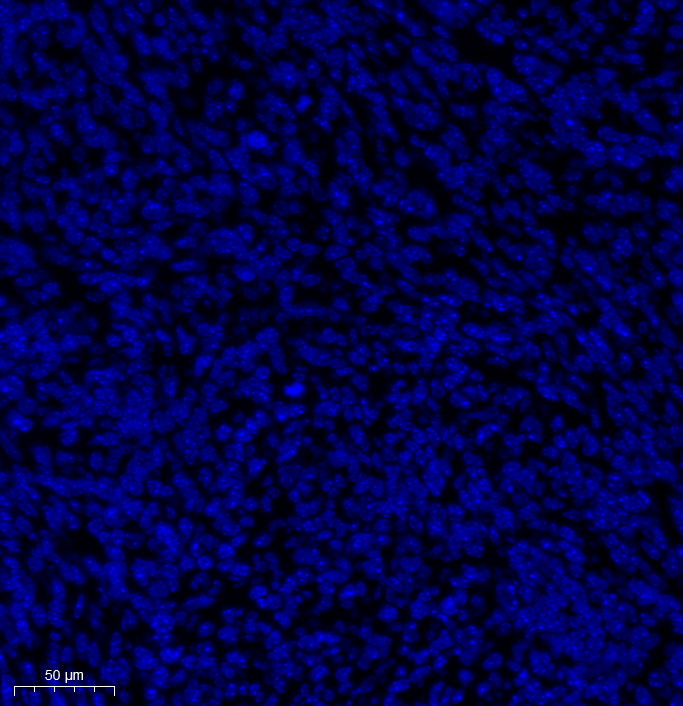

Supplement: Supplementary file 1 [file DataSheet_1.zip › rawdata-IF/DEPTOR/Tumor/T-DAPI.jpg]

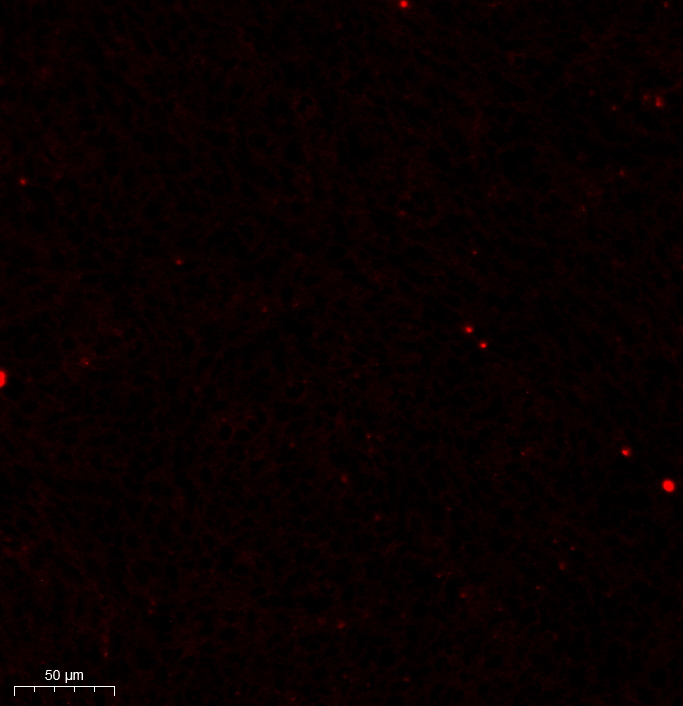

Supplement: Supplementary file 1 [file DataSheet_1.zip › rawdata-IF/DEPTOR/Tumor/T-DEPTOR.jpg]

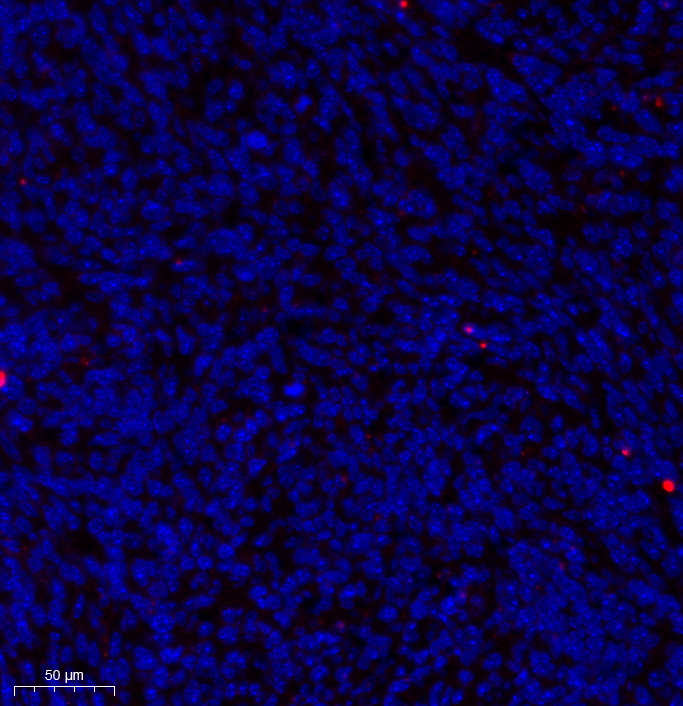

Supplement: Supplementary file 1 [file DataSheet_1.zip › rawdata-IF/DEPTOR/Tumor/T-merge.jpg]

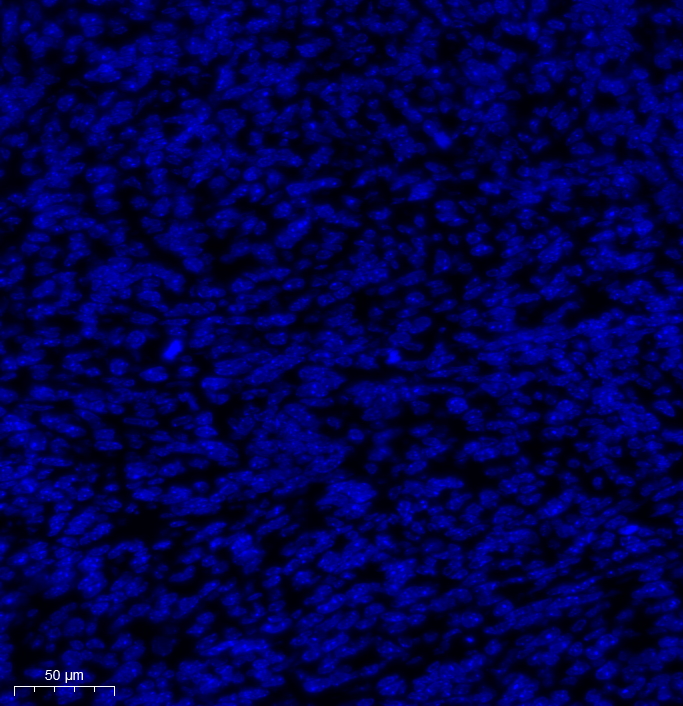

Supplement: Supplementary file 1 [file DataSheet_1.zip › rawdata-IF/SPAG4/Nc/NC-DAPI.jpg]

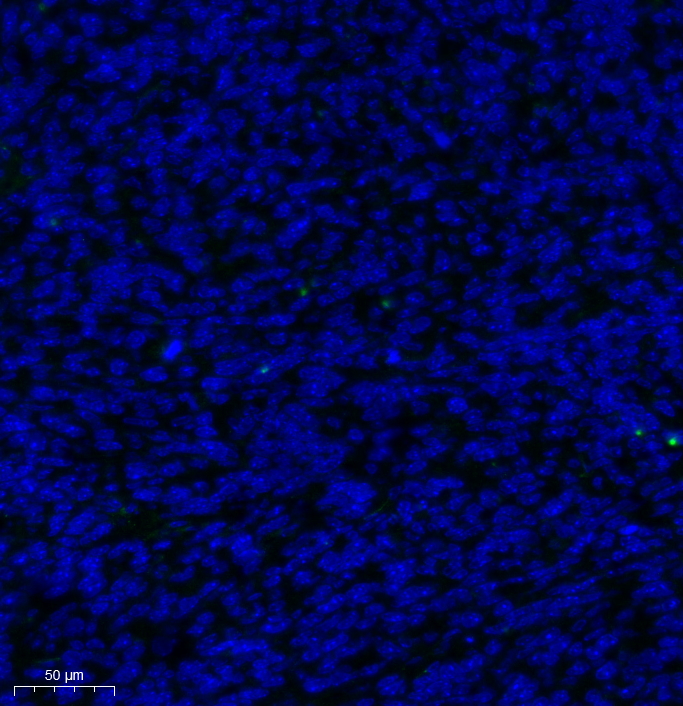

Supplement: Supplementary file 1 [file DataSheet_1.zip › rawdata-IF/SPAG4/Nc/Nc-merge.jpg]

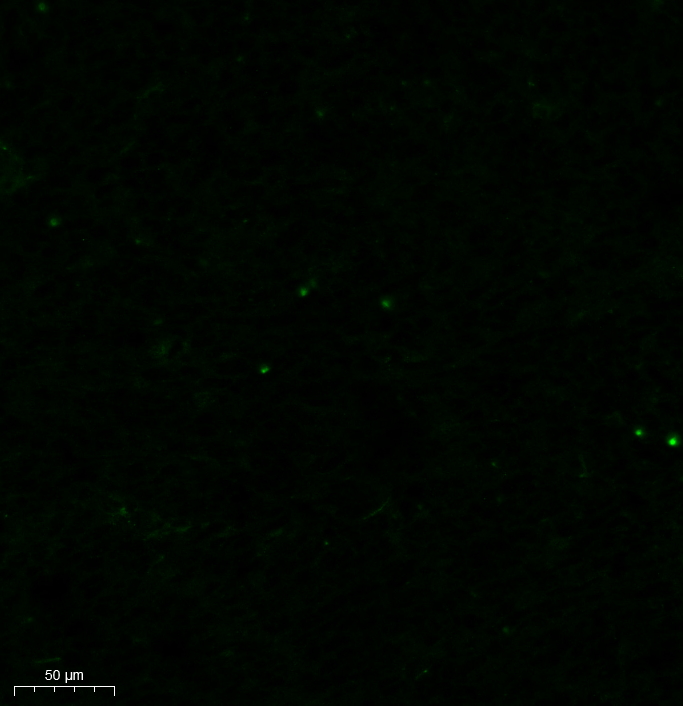

Supplement: Supplementary file 1 [file DataSheet_1.zip › rawdata-IF/SPAG4/Nc/NC-SPAG4.jpg]

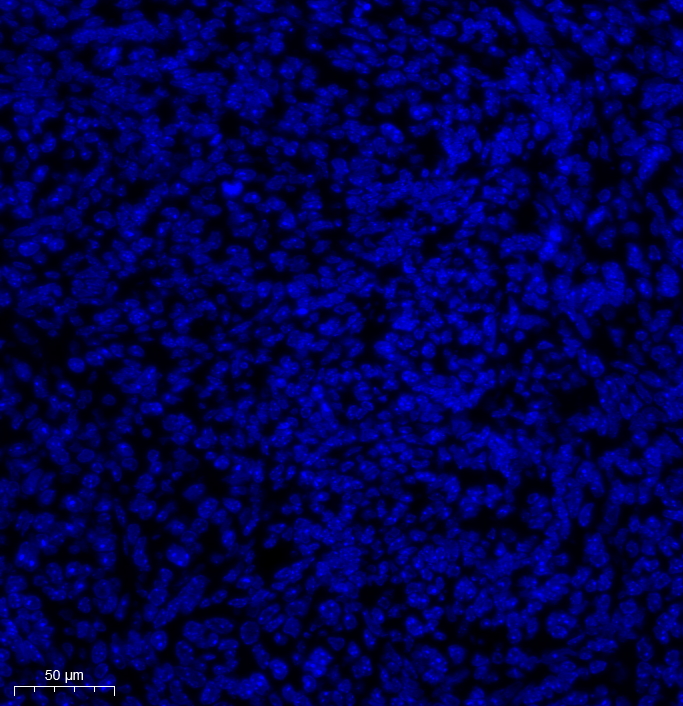

Supplement: Supplementary file 1 [file DataSheet_1.zip › rawdata-IF/SPAG4/T/T-DAPI.jpg]

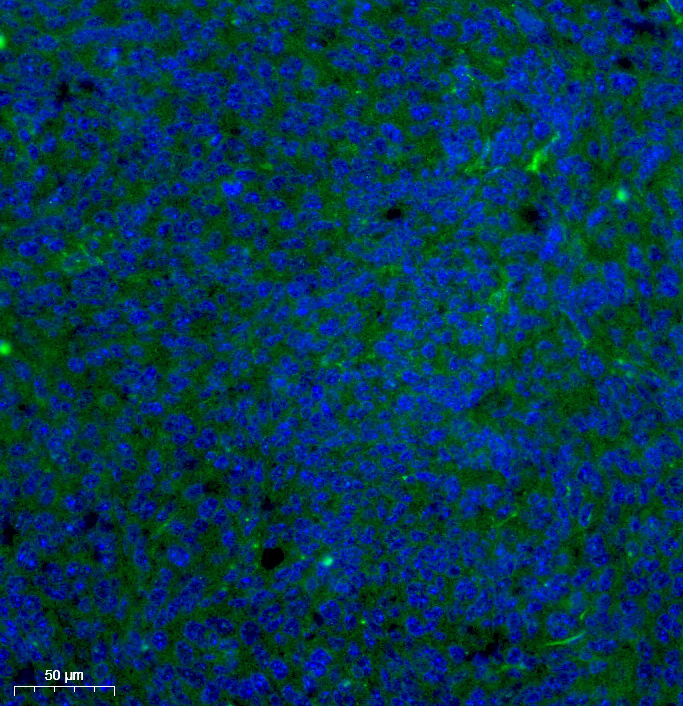

Supplement: Supplementary file 1 [file DataSheet_1.zip › rawdata-IF/SPAG4/T/T-merge.jpg]

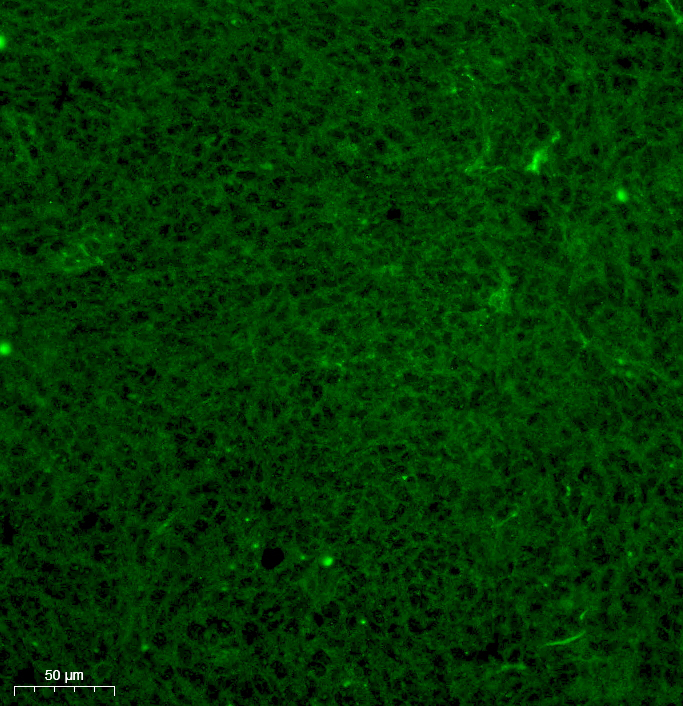

Supplement: Supplementary file 1 [file DataSheet_1.zip › rawdata-IF/SPAG4/T/T-SPAG4.jpg]

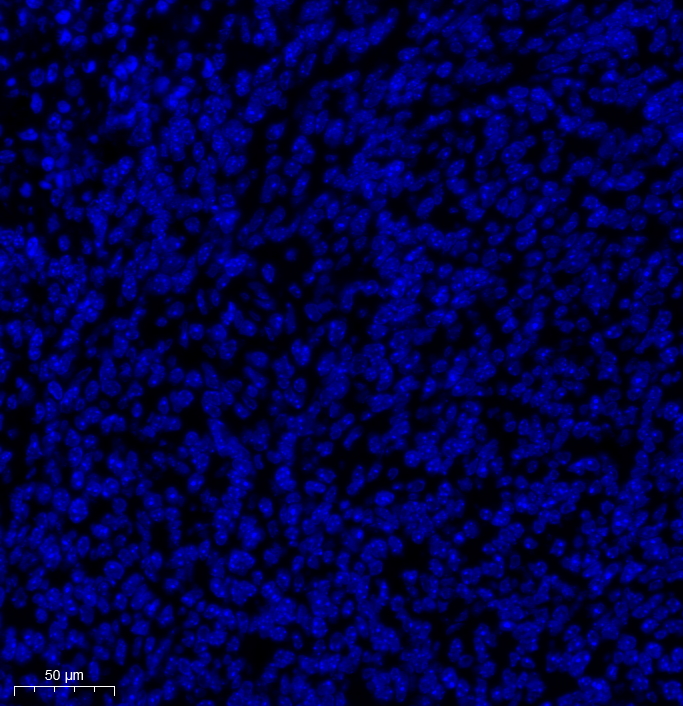

Supplement: Supplementary file 1 [file DataSheet_1.zip › rawdata-IF/ZBTB32/Nc/Nc-DAPI.jpg]

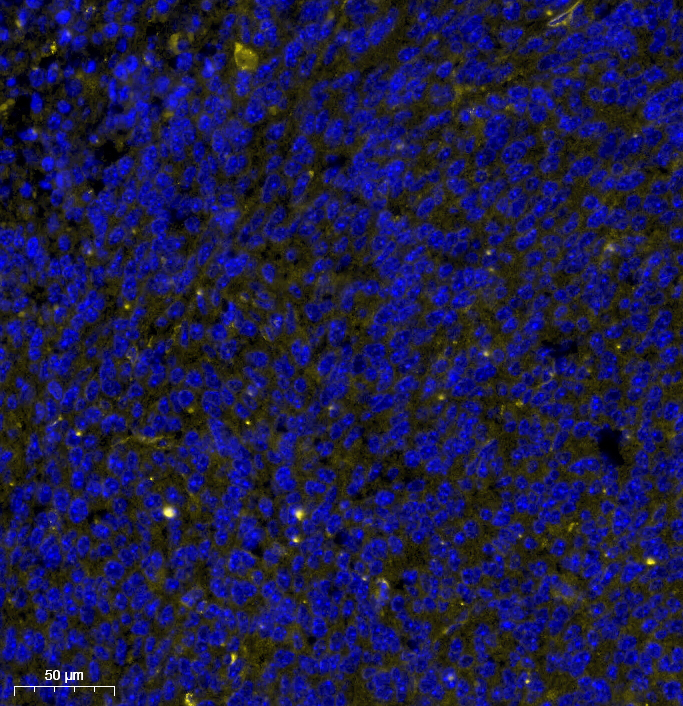

Supplement: Supplementary file 1 [file DataSheet_1.zip › rawdata-IF/ZBTB32/Nc/Nc-merge.jpg]

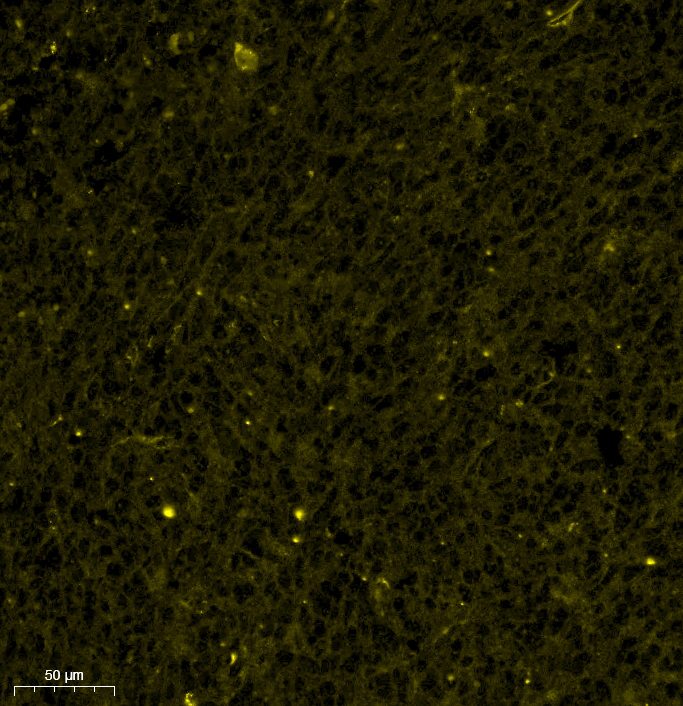

Supplement: Supplementary file 1 [file DataSheet_1.zip › rawdata-IF/ZBTB32/Nc/Nc-ZBTB32.jpg]

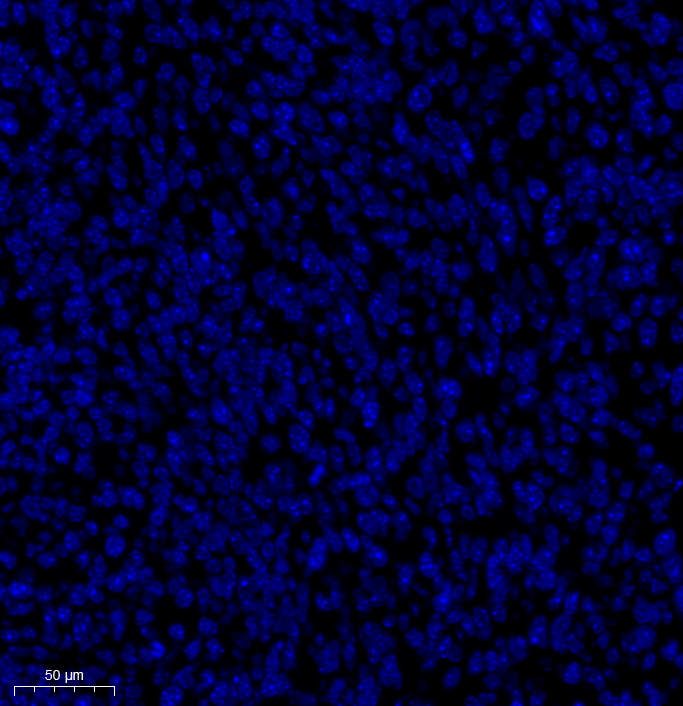

Supplement: Supplementary file 1 [file DataSheet_1.zip › rawdata-IF/ZBTB32/T/T-DAPI.jpg]

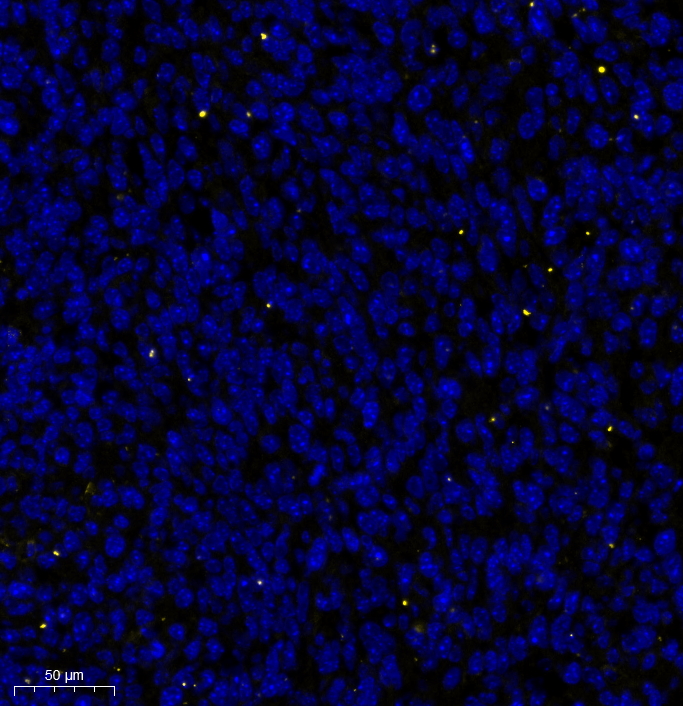

Supplement: Supplementary file 1 [file DataSheet_1.zip › rawdata-IF/ZBTB32/T/T-merge.jpg]

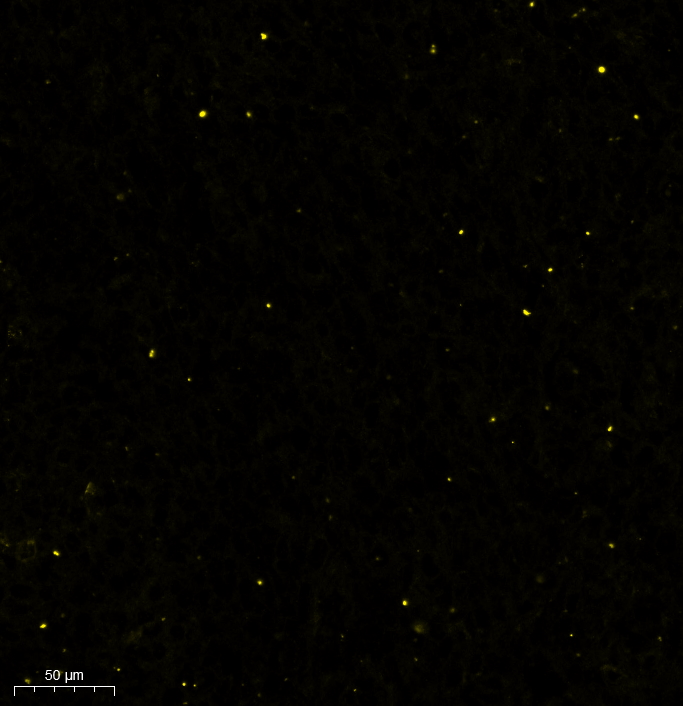

Supplement: Supplementary file 1 [file DataSheet_1.zip › rawdata-IF/ZBTB32/T/T-ZBTB32.jpg]

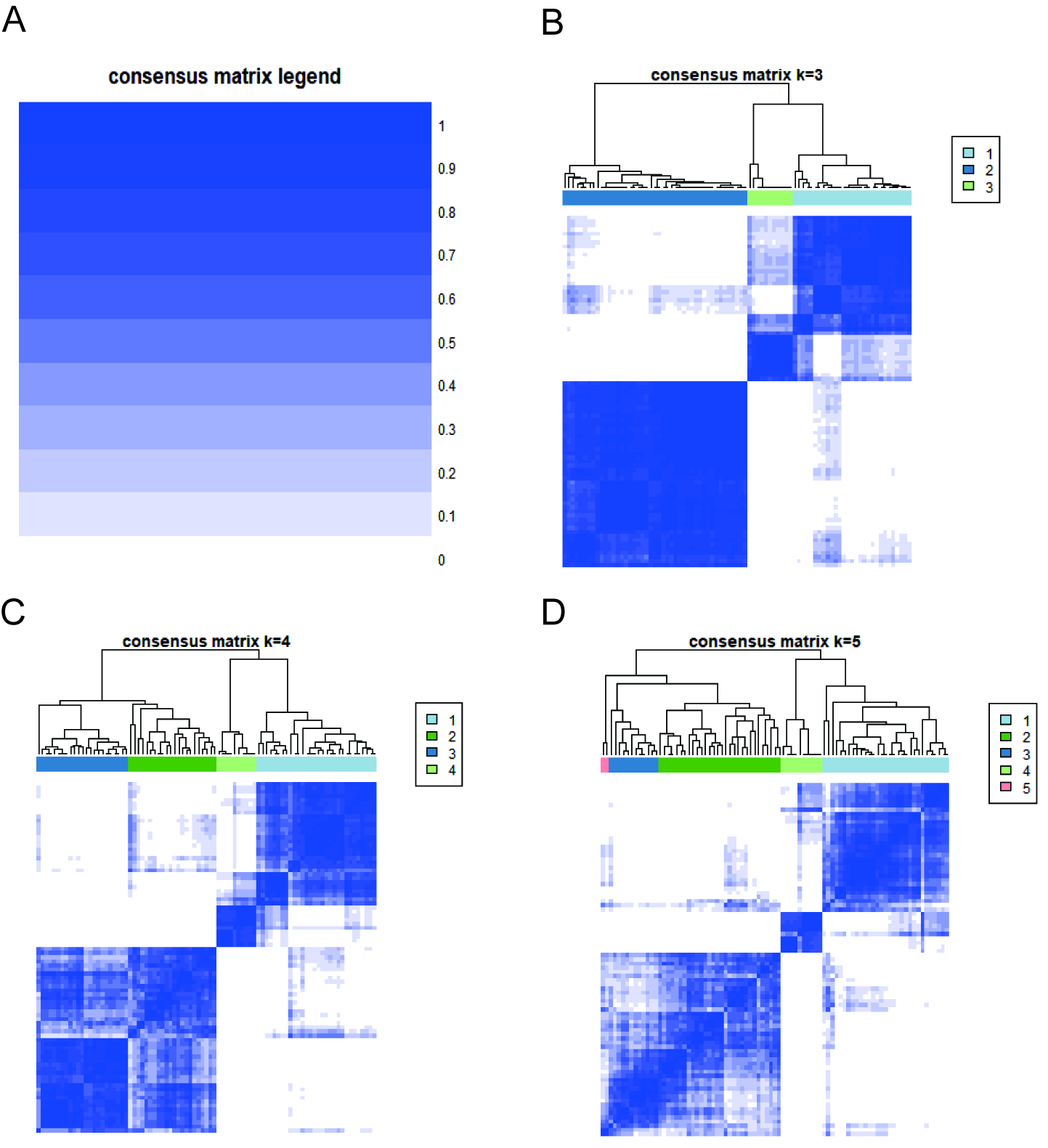

Supplement: Supplementary file 3 [file Image_1.tif]

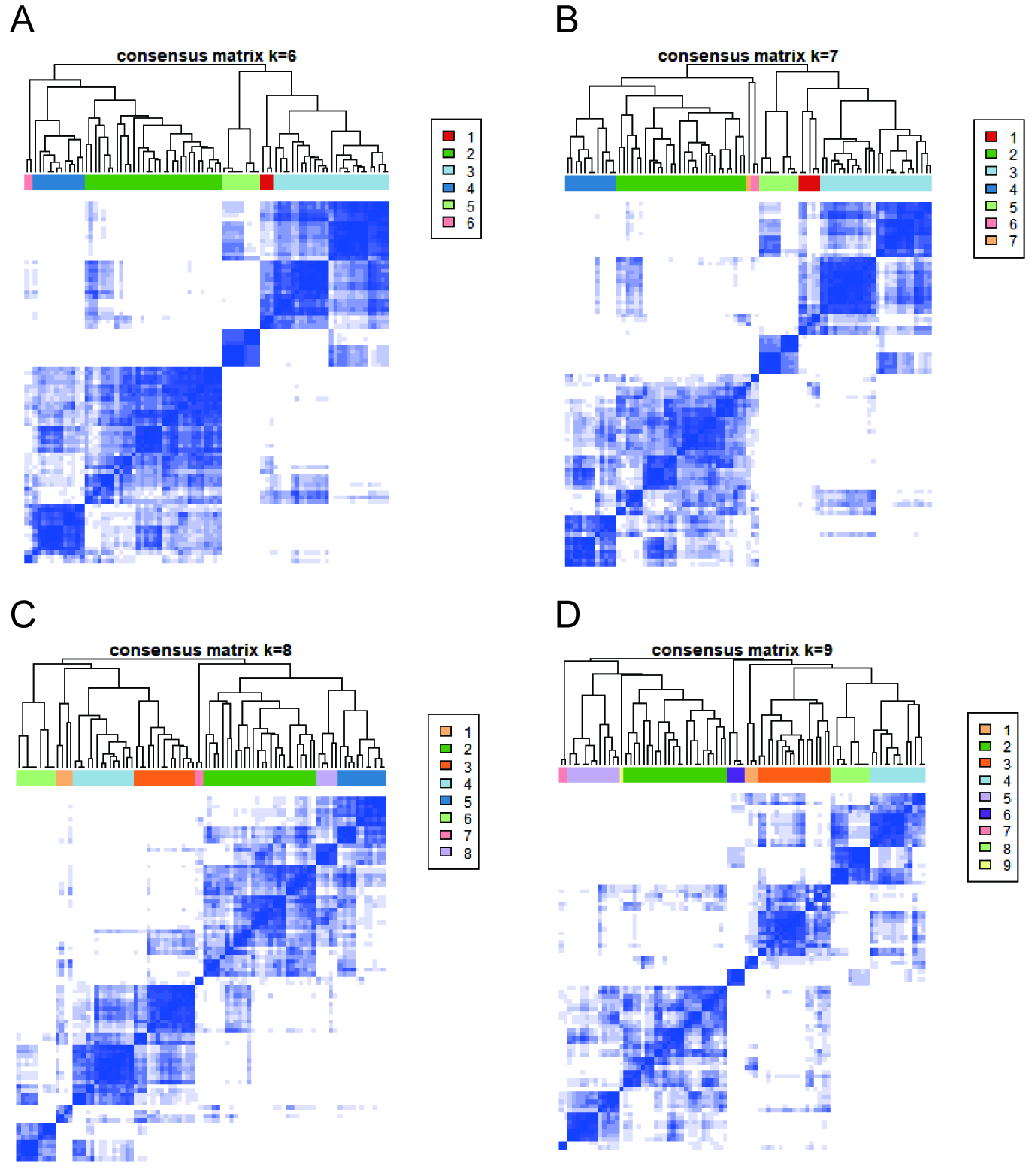

Supplement: Supplementary file 4 [file Image_2.tif]

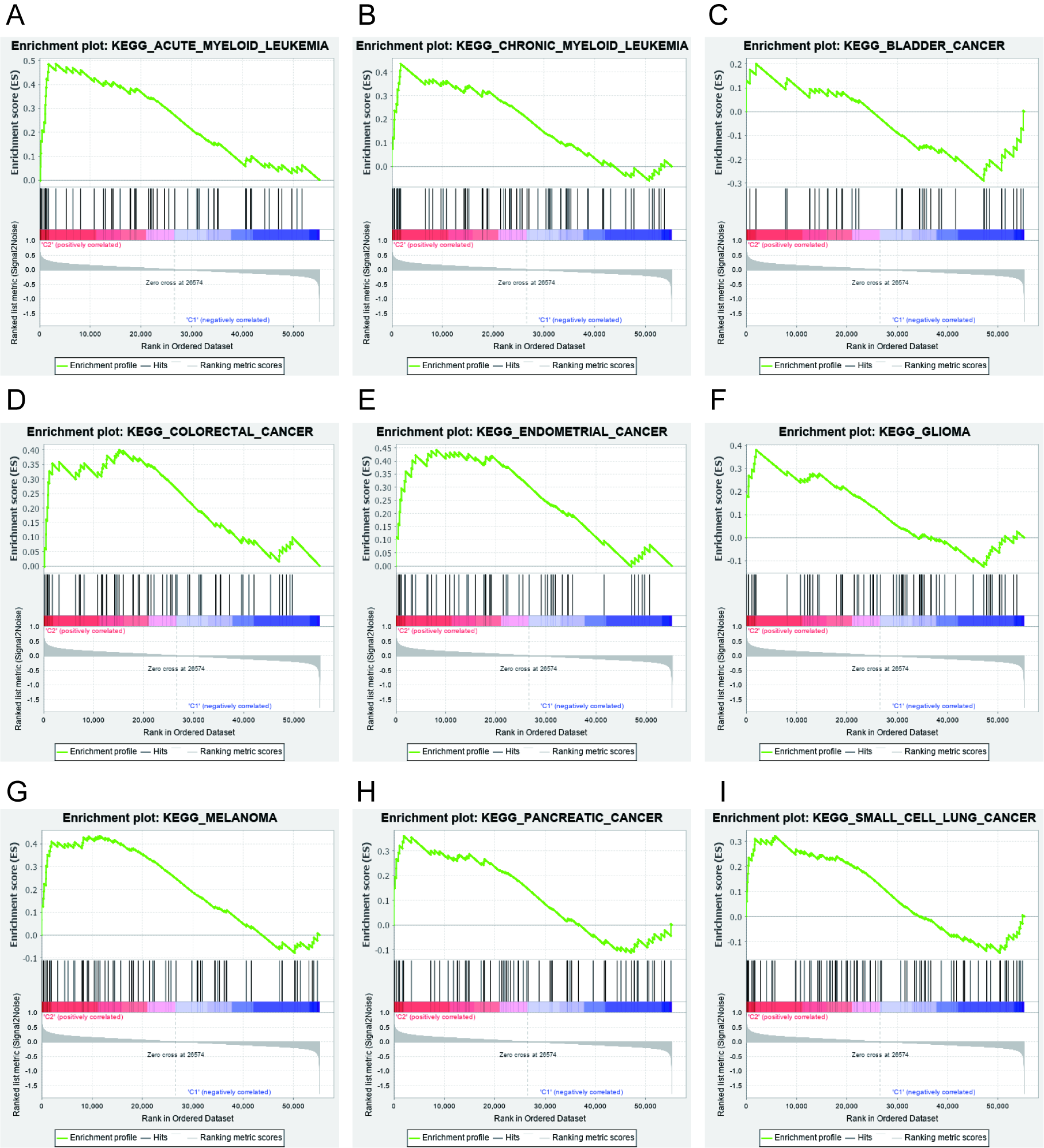

Supplement: Supplementary file 5 [file Image_3.tif]

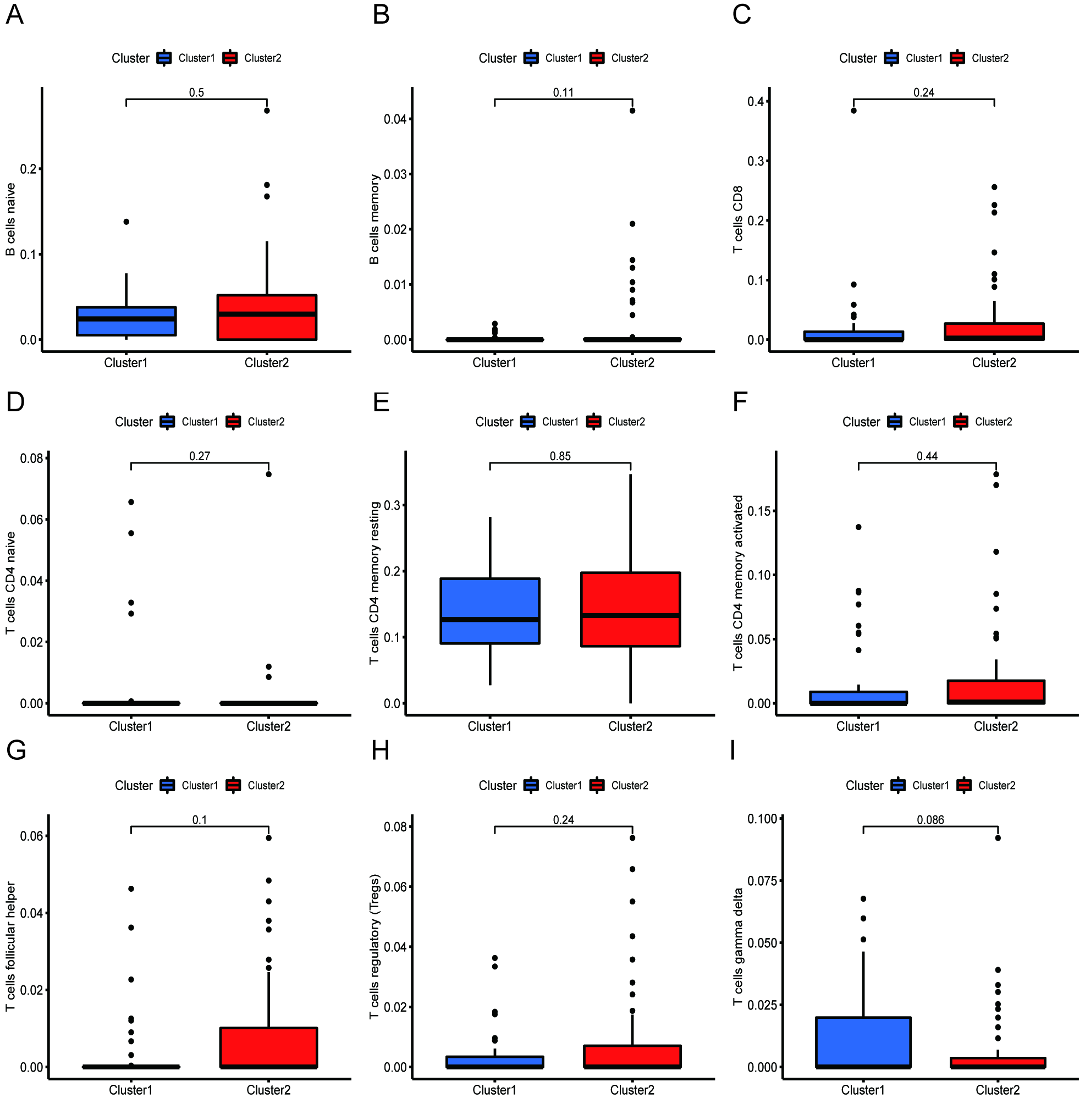

Supplement: Supplementary file 6 [file Image_4.tif]

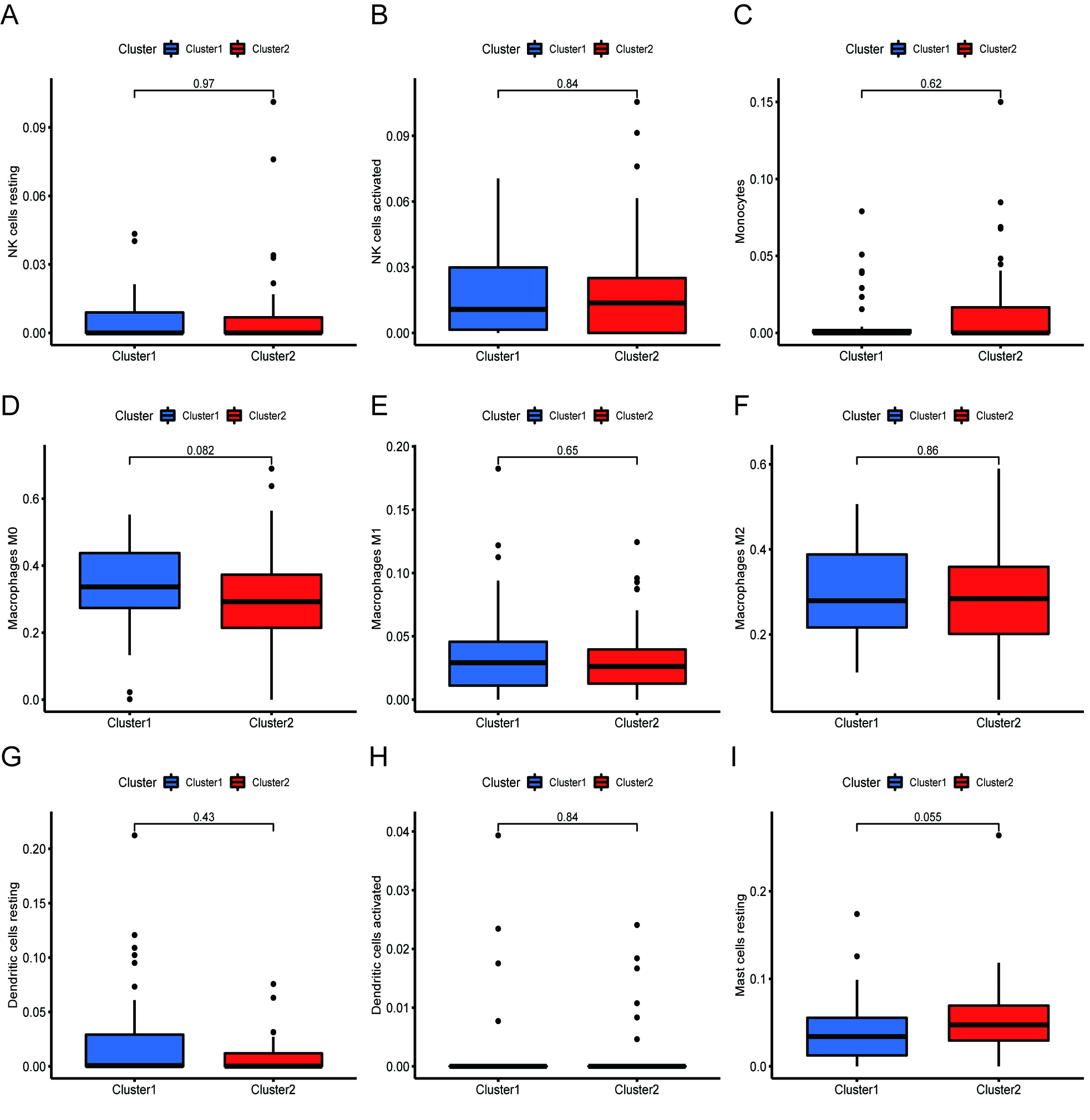

Supplement: Supplementary file 7 [file Image_5.tif]

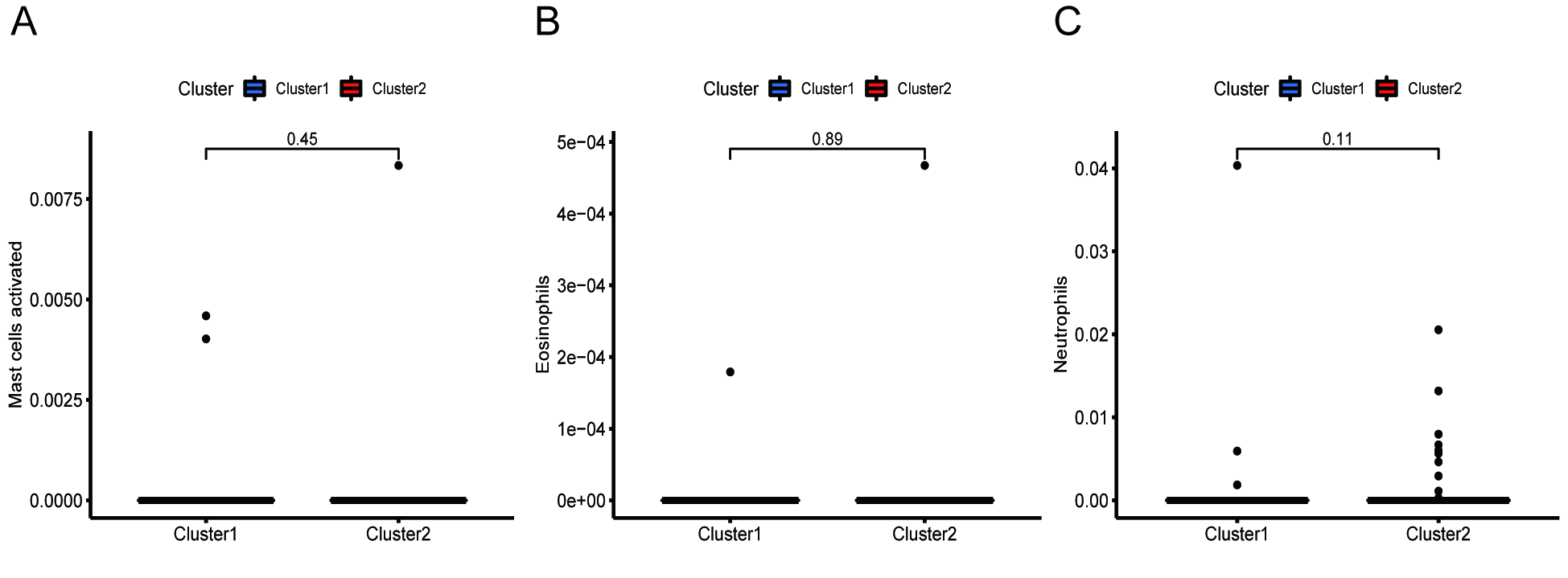

Supplement: Supplementary file 8 [file Image_6.tif]
